# Nonlinear Optical Activity of a Chiral Organic–Inorganic ([(NH_3_CH_2_CH_2_)_3_NH])_2_[MnBr_5_]Br_5_ Photoluminescent and Piezoelectric Crystal

**DOI:** 10.1021/acs.jpclett.4c00709

**Published:** 2024-05-09

**Authors:** Magdalena Rok, Andrzej Miniewicz, Maria Zdończyk, Bartosz Zarychta, Julia W. Mikurenda, Stanisław Bartkiewicz, Monika Wiśniewska-Bełej, Joanna Cybińska, Anna Piecha-Bisiorek

**Affiliations:** † Faculty of Chemistry, University of Wroclaw, 14 F. Joliot - Curie, 50-383 Wroclaw, Poland; ‡ Institute of Advanced Materials, Faculty of Chemistry, Wroclaw University of Science and Technology, Wybrzeże Wyspiańskiego 27, 50-370 Wroclaw, Poland; § Łukasiewicz Research Network - PORT Polish Center for Technology Development, ul. Stabłowicka 147, 54-066 Wrocław, Poland; ∥ Faculty of Chemistry, University of Opole, Oleska 48, 45-052 Opole, Poland

## Abstract

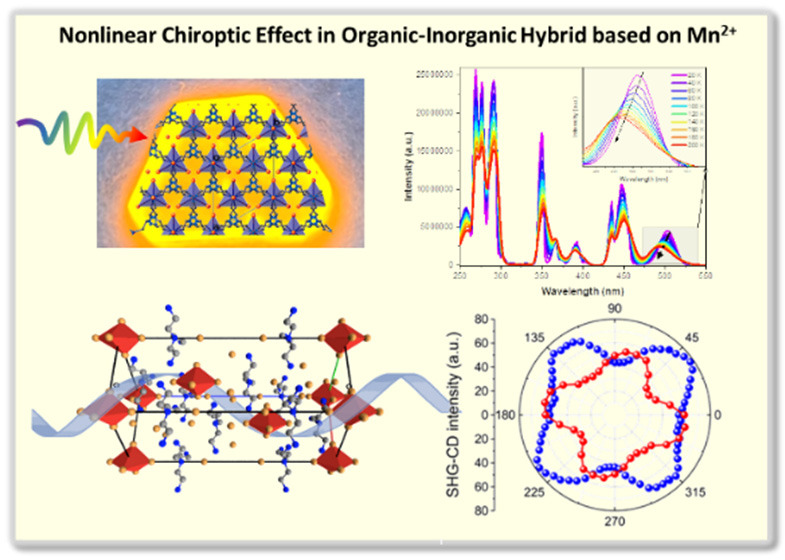

The family of Mn-based organic–inorganic hybrids has greatly expanded due to their advantages in applications. They also show superior bright and size-tunable photoluminescence and can be considered a perfect alternative to toxic lead-based compounds. In this work, we present the detailed structural, optical, and electrical characterization of ([(NH_3_CH_2_CH_2_)_3_NH])_2_[MnBr_5_]Br_5_. The title compound exhibits a unique type of inorganic arrangement created by the trigonal bipyramids. It crystallizes in noncentrosymmetric space group *R*32, indicating its optical activity, piezoelectricity, and second-order optical nonlinearity proven by the second harmonic of light measurements. The studied crystals exhibit intense photoluminescence originating from the Mn(II) ion ^4^T_1_(G) → ^6^A_1_ transition. The measured lifetime of the photoluminescence emission is ≤1.5 ms, while the measured quantum yield for both powder and crystal samples reaches ∼70%.

Currently, materials, especially organic–inorganic hybrids (OIHs), that exhibit functional properties entirely different from those of the starting substrates are being sought. This is due to their key features, such as easy and inexpensive synthesis in solution, high mechanical flexibility, structural tunability, and chemical diversity, to name a few. Additionally, the organic–inorganic combination allows one to obtain stimulus-responsive materials sensitive to the changing external environment, such as an electric field (alternating or direct current), pressure, temperature, or polarized light. Considering lead toxicity, developing highly stable, lead-free metal halide materials is crucial for basic scientific research and sustainable technological development. Among the environmentally friendly hybrids employing diverse metal cations [Sn(II), Cu(II), Ag(I), Bi(III), Sb(III), and In(III)]^1−3^ and the recently reported chiral hybrids of Ge(II)^4^ and Ce(II),^5^ the Mn(II)-based compounds appear to be an auspicious choice. The attractiveness mentioned above is related to the electron structure of transition cation Mn^2+^, whose valence electrons (3d^5^) are exposed in the outer layer, and the position of the emission band strongly depends on the surrounding Mn(II) cation crystal field. Halide ligands produce a weak electric field and thus have little effect on the splitting of d orbitals in the crystal field, making the complexes formed high-spin complexes. Mn(II) cations with halide ligands typically form organic–inorganic complexes with an octahedral (*O*_*h*_) configuration (with stoichiometry ABX_3_) or tetrahedral (*T*_*d*_) complexes with the general formula A_2_BX_4_. On the basis of the structures deposited in the Cambridge Structural Database, only a few crystals possess polar properties in octahedral or tetrahedral configurations. The noncentrosymmetric character of some crystals has been proven by measuring the ferroelectric hysteresis loop,^6−12^ the piezoelectric effect,^12,13^ or the nonlinear optical (NLO) activity.^7,9,14−17^ An additional attraction of the Mn(II) OIHs stems from the photoluminescent properties of the crystals, where the emission color depends on the coordination environment of the Mn(II). Typically, octahedral Mn(II) coordination shows orange to red emission,^11,16−19^ primarily due to the relatively robust crystal field strength. The intense green photoluminescence emission^8,19−23^ originates from the absence of an inversion center of the *T*_*d*_ environment, and the small crystal field splitting energy of [MnX_4_]^2–^ (where X denotes a halogen atom) increases the transition dipole moment oscillator strength. Dual-color emission also exists when both coordination environments are present. Such an example was discussed for the inverted perovskite structure system, [(CH_3_)_3_NH]_3_(MnBr_3_)(MnBr_4_).^24^ This opens the possibility of developing materials with well-defined luminescent properties that strictly depend on their crystal structure, thus allowing the design and tailoring of these properties. It should be noted that, for example, Mn^2+^ ion-doped materials that exhibit efficient red emission can be promising substitutes for the widely used Eu(III) ions.^25^

There is limited literature on the physicochemical properties of Mn(II) complexes with coordination different from octahedral or tetrahedral coordination. In 2017, Mei et al.^26^ described a unique example of a coordinated Mn(II) cation with a halide anion, where the Mn(II) cation adopted a trigonal bipyramidal geometry in the [MnBr_5_]^3–^ unit. However, few reports have presented the trigonal bipyramidal symmetry of Mn(III) complexes to date.^27−30^ Another example of the trigonal bipyramid was obtained in a crystal for which L = 1-(cyclopentyl)-4-aza-1-azoniabicyclo[2.2.2]octane. However, in this compound, the organic part coordinates with the inorganic units, creating fairly complex dimers containing two Mn–Cl–Mn bridges.^31^ This indicates that the trigonal bipyramidal geometry of Mn(II) is extremely rare and worthy of more in-depth analysis. In the complex in question, organic cation ((NH_3_CH_2_CH_2_)_3_NH)^4+^ and two uncoordinated Br^–^ anions were linked by hydrogen bonds to form cavities in which the [MnBr_5_]^3–^ unit was enclosed. (C_6_N_4_H_22_)_2_[MnBr_5_]Br_5_, hereafter **TMB**, crystallizes in trigonal noncentrosymmetric space group *R*32, and this is an excellent example of a chiral hybrid organic–inorganic structure that indicates its optical activity, piezoelectricity, and nonlinear chiroptical properties. The chiroptical and chirally related electric and magnetic properties have recently attracted a great deal of attention due to both scientific interest and potential applications in various fields (see the recently published review publications and references therein).^32−36^ Long et al.^32^ in the excellent review concerning the research and applications of chiral perovskites in optoelectronics give a broad outlook on different aspects of these very promising materials. Among the unique achievements, one can mention reports on spin control in reduced-dimensional chiral perovskites,^37^ their topological quantum properties,^38^ spintronics, a report on chiral perovskite photodetectors with responsivity 100 times higher than that of chiral metasurface photodetectors,^39,40^ and the third-order nonlinear optical effect of two-photon absorption upconverted circularly polarized fluorescence in chiral perovskite nanocrystals,^40^ to mention just a few.

Here, we report on the first measurements of **TMB** crystal circular dichroism (CD), second harmonic generation circular difference (SHG-CD, known, improperly, also as SHG-circular dichroism), and luminescence properties in a wide temperature range. Ultraviolet–visible (UV–vis) spectra, fluorescence decay lifetimes, and photoluminescence quantum yields (PLQYs) were measured with correlation to the crystal structure. We report also on dielectric measurements as a function of temperature, frequency, and crystal stability.

**TMB** crystals were obtained by a simple reaction of tris(2-aminoethyl)amine and MnBr_2_·4H_2_O with a stoichiometry ratio of 1:1 in an aqueous solution of HBr acid. Via slow evaporation at room temperature, yellow single crystals of **TMB** were obtained (see Figure 1). Powder X-ray diffraction verified the phase purity (see Figure S1).

**Figure 1:**
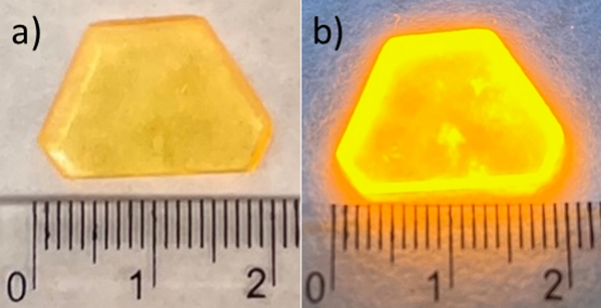
Single crystals of **TMB** crystallized from the aqueous solution: (a) as observed under ambient light and (b) under UV light, showing intense yellow luminescence.

According to the thermal analysis shown in Figure S2, **TMB** crystals are thermally stable up to 500 K. Above this temperature, melting with decomposition occurs. In the range of 100–500 K, no phase transformations were observed. Notably, the crystal exposed to air at room temperature does not exhibit hygroscopic properties.

The structure of **TMB** has been measured at 100 K (crystal data summarized in Table S1), and the asymmetric part of the unit is shown in Figure 2. Geometrical parameters and hydrogen bond geometry are summarized in Tables S2 and S3.

**Figure 2:**
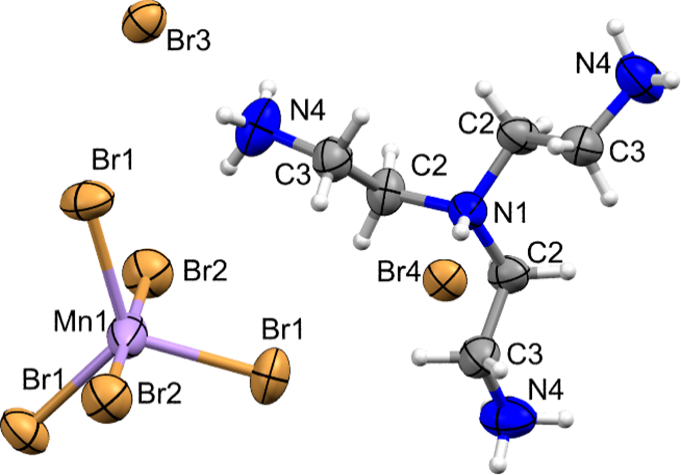
Crystal structure of **TMB** in an asymmetric part of the unit. Anisotropic ellipsoids are drawn at the 99% probability level. Hydrogen atom labels and symmetry equivalents of Br3 and Br4 have been omitted for the sake of clarity.

The structure of **TMB** was previously reported at 123 K.^26^**TMB** crystallizes in the high-symmetry, chiral space group of *R*32. Note that the chirality of this compound is neither imparted by incorporation of any chiral organic molecules nor induced by tuning the environmental conditions, e.g., by using chiral solvents^41^ or external stimuli^42^ (such as strain). The unit cell is composed of Mn(II) cations coordinated by five bromide anions in a trigonal–bipyramidal arrangement, tetraprotonated tris(2-ammonioethyl)ammonium (H_4_*tren*) cations, and five noncovalently bonded bromide anions as counterions (two of them are symmetrically independent). The crystal packing comprises a multilayer arrangement of inorganic ([MnBr_5_]^3–^) moieties, nonchiral organic [tris(2-ammonioethyl)ammonium] cations, and inorganic Br3/Br4 in two distinct sheets forming a cake-like structure (Figure 3).

**Figure 3:**
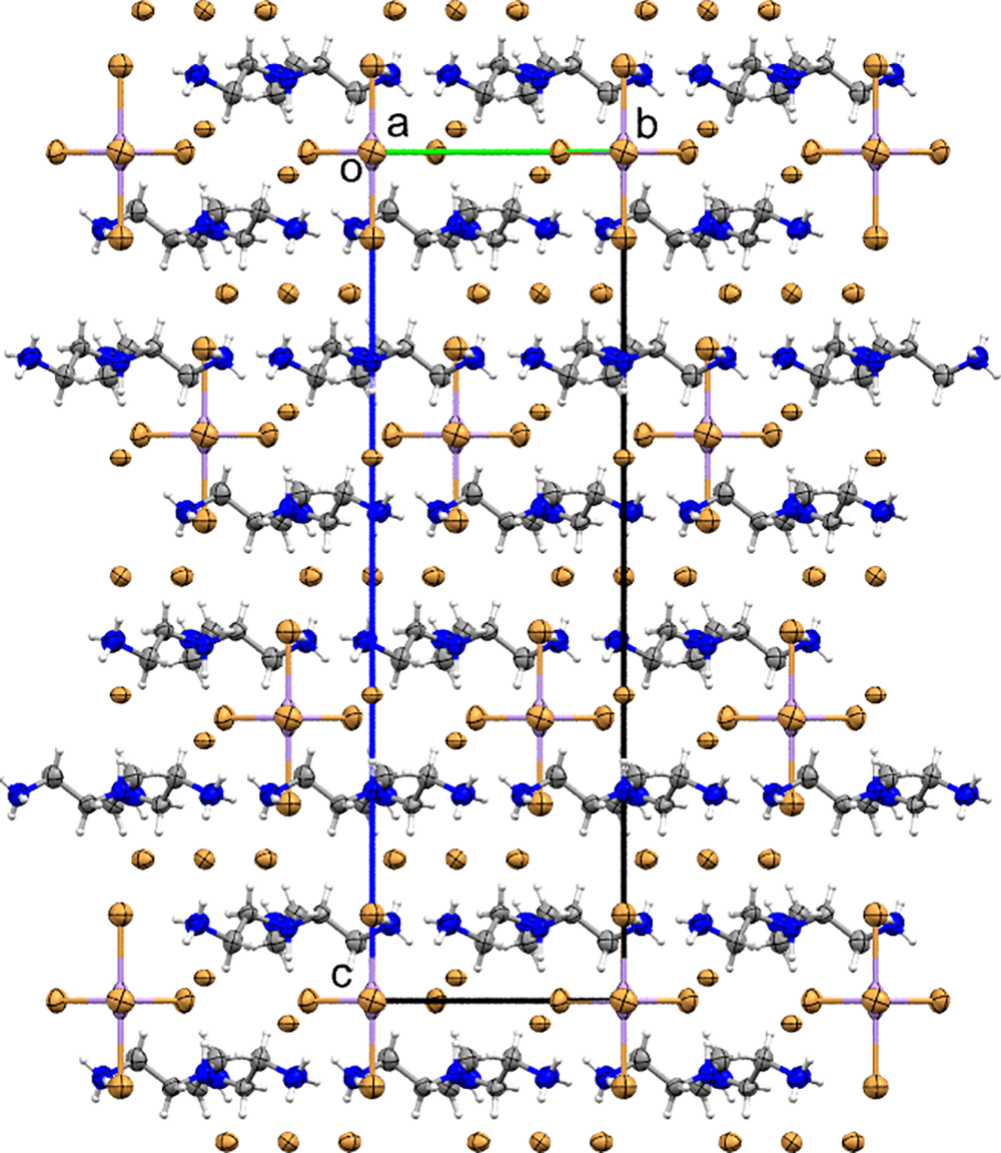
Packing diagram of **TMB** viewed along the *a* direction. Anisotropic ellipsoids are drawn at the 99% probability level.

The layers are arranged parallel to the (001) plane and are connected to each other by rather weak but numerous N–H···Br hydrogen bonds and cation–anion interactions (Table S3). The strongest [shortest and linear with a ∠N–H···Br angle of 180(1)°] interaction occurs between organic cations and the Br4 layer. Moreover, this assembly also holds the [MnBr_5_]^3–^ bipyramids in its voids. The elongated trigonal bipyramidal arrangement of the [MnBr_5_]^3–^ moiety is highly symmetric as it has a Mn(II) cation at a 3a Wyckoff position (32 symmetry) and two symmetrically independent bromide atoms at 9d (side symmetry of 2, equatorial Br1 atom) and 6c (side symmetry of 3, axial Br2). The difference between two distinct Mn–Br bonds is somehow significant [Δ = 0.422(1) Å], especially when the experimental magnetic moment is considered (6.47 μ_B_), which indicates the equal distribution of unpaired electrons within d orbitals in the high-spin configuration. The trifurcated short contact between Br2 and H2*B* atoms accompanied by electrostatic interaction [the Br2 atom points directly to the center of the H_4_*tren* cation; Br2···N1, 4.18(1) Å] may play a crucial role in such distortion (Table S3). The H_4_*tren* cation adopts a stretched conformation with a N_c_–N_t_ distance of 3.779(3) Å. The molecule is highly symmetric as the central N1 atom occupies the 6c Wyckoff position (side symmetry of 3) with a N_t_–N_c_–N_t_ angle of 119.8(2)°. The remaining bond distances and angles within the molecule are typical.

The chirality of the *R*32 space group is realized here by the framework arrangement, as none of the unit cell contents is chiral. The trigonal, noncentrosymmetric structure of the H_4_*tren* cation plays a crucial role in the assembly of the chiral crystal. The [MnBr_5_]^3–^ anions arranged by N–H···Br hydrogen bonds form a “corkscrew” motif in the *c* direction, as depicted in Figure 4.

**Figure 4:**
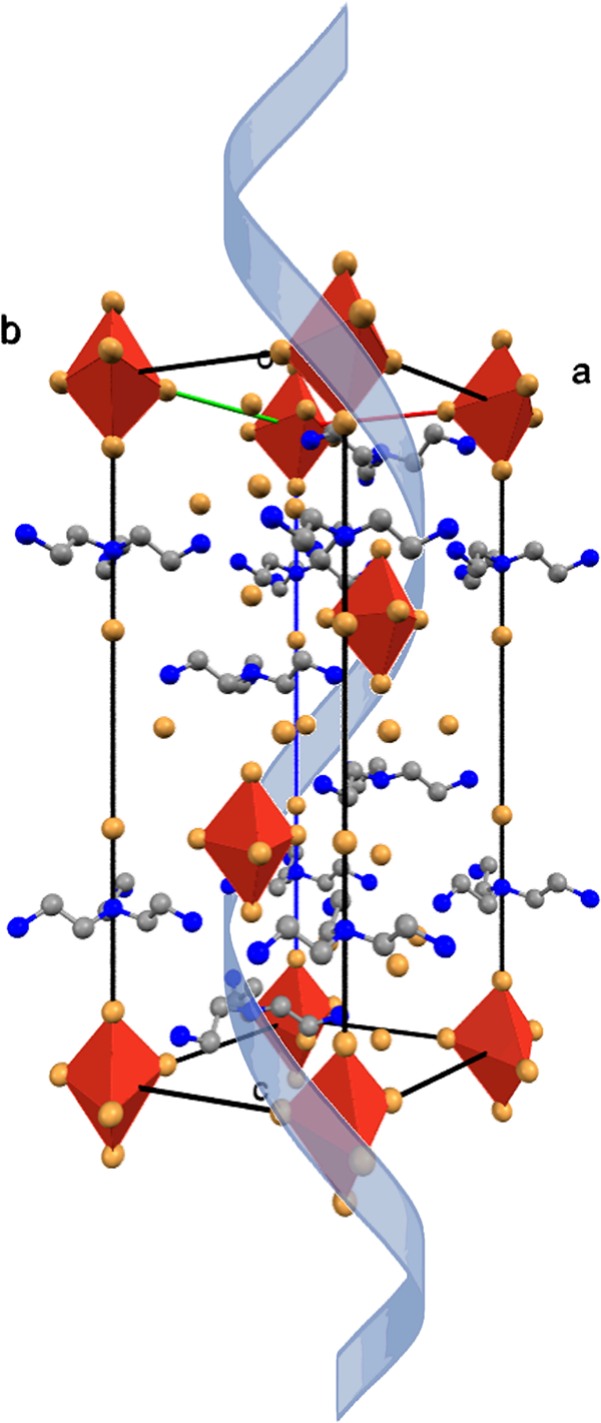
“Corkscrew” motif in the arrangement of [MnBr_5_]^3–^ trigonal bipyramids around the 3_1_ screw axis.

The **TMB** structure is stabilized by the intermolecular hydrogen bonds and electrostatic van der Waals interactions.

Panels a and b of Figure 5 show the dielectric response, which was measured on a monocrystalline sample (see also chapter 1.3 of the Supporting Information). The crystal was crystallographically oriented perpendicular to the *a–b* plane, and silver paste electrodes were applied to the surfaces. During cooling, the real part of the electric permittivity (ε′) increases slightly with a decrease in temperature. Along the *c* axis (perpendicular to the largest crystal surface), dispersive (ε′) and absorptive (ε″) behavior was not observed. This indicates the absence of dielectric relaxation processes in the analyzed frequency range between 135 Hz and 2 MHz. In other words, the negligible dynamics of dipolar molecules arise in the crystal. Also, analyzing the imaginary part of the electric permittivity dependence on temperature [ε″(*T*)], we can conclude that there is no additional contribution to the dielectric response associated with ac conductivity. In contrast, a contribution related to a more desirable property than dielectric relaxation is observed. The characteristic resonance curves appearing on the ε′(*T*) dependence curve at the experimental frequencies of 133.3, 456.4, 583.9, 746.9, and 1563 kHz and related absorption anomalies for a 1.4 mm thick **TMB** sample are typical for crystals lacking the center of symmetry and are the result of the contribution of piezoelectric resonances to electric permittivity. The appearance of these anomalies over a wide range of temperatures testifies indirectly to the piezoelectric properties of the **TMB** crystal. We simply observe that at certain crystal temperatures the used frequency of the ac electric field matches perfectly with the mechanical crystal resonance [local ε′(*T*)_max_] followed by the associated antiresonance [local ε′(*T*)_min_], as clearly shown in Figure 5a. Additional information about the piezoelectric effect in chiral space group *R*32 is available in chapter 1.3.2 of the Supporting Information.

**Figure 5:**
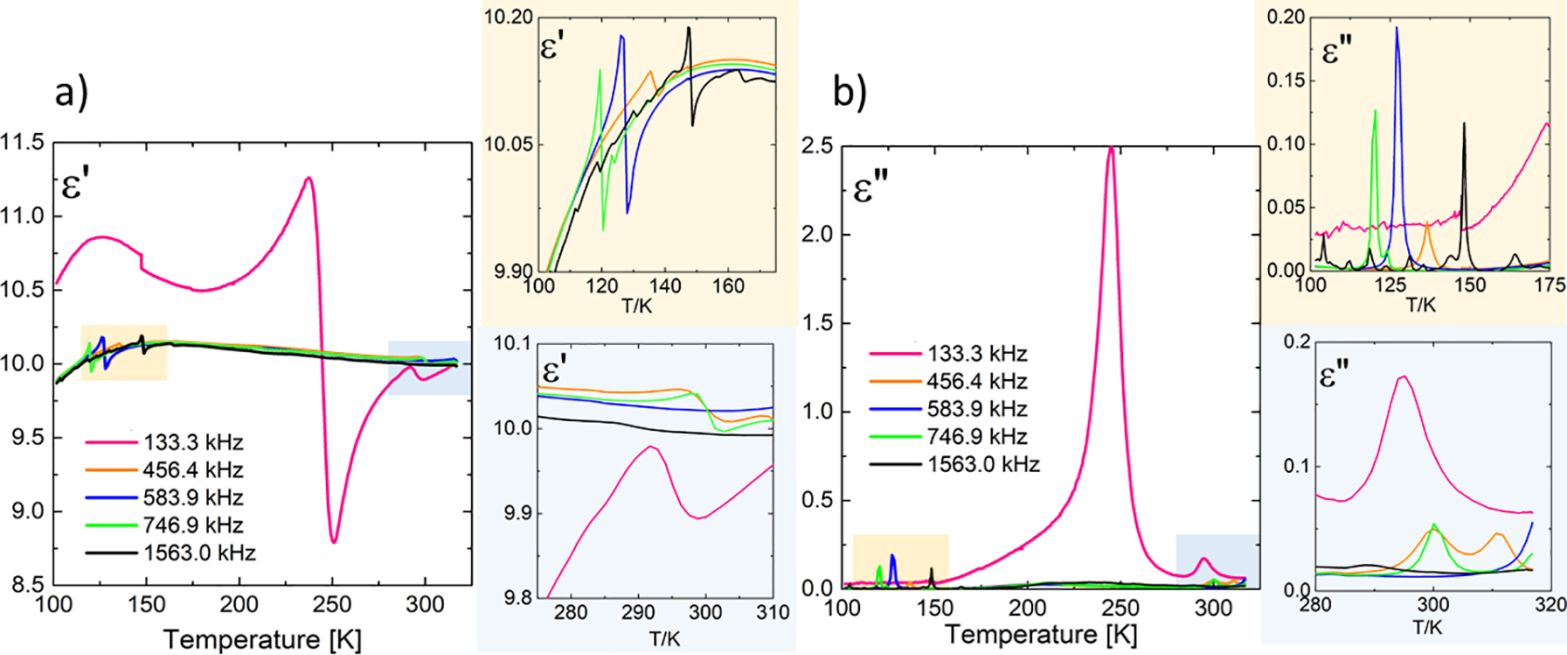
Dielectric response of the **TMB** single crystal at different frequencies as a function of temperature, (a) ε′(*T*) and (b) ε″(*T*), with resonant piezoelectric contributions measured along the *c* axis (*S* = 70 mm^2^; *d* = 1.40 mm).

The most interesting properties related to materials crystallizing in one of the 65 Sohncke space groups^43−45^ are related to chiroptical ones. The crystals belonging to trigonal space group *R*32 (No. 155) can exhibit piezoelectricity but not pyroelectricity or ferroelectricity due to the lack of a polar axis. However, the optical activity and second-order optical nonlinearity effects related to chirality are allowed. The *R*32 symmetry predicts that the **TMB** crystal is optically uniaxial, i.e., described by a rotational ellipsoid of refractive indices *n*_e_ and *n*_o_. Its chirality indicates that complex indices of refraction [*n*_c_(λ) = *n′*(λ) + *in*″(λ)] should be different for left- and right-handed circularly polarized light (LCP and RCP, respectively) directed along the chiral *c* axis. The linear optical activity called optical rotation (OR) (also termed circular birefringence) is linked with a real part *n*′, while circular dichroism (CD) is linked with an imaginary part of refractive index *n*″.

CD is the key example of chiral light–matter interaction, being extensively exploited as a spectroscopic probe of the chirality of compounds and supramolecular aggregates.^46−49^ The anisotropy factor related to excitonic effects [*g*_CD_ ≡ 2(*A*_L_ – *A*_R_)/(*A*_L_ + *A*_R_)] is usually small, i.e., in the range of 10^–4^ to 10^–2^, where *A*_L_ and *A*_R_ are the left- and right-handed circularly polarized absorbance, respectively. CD is sensitive to chirality in energy transitions. The extinction coefficients for LCP [ε^L^(λ)] and RCP [ε^R^(λ)] light are different; therefore, upon exiting the sample, the electric field of the transmitted wave describes an ellipse. The amount of ellipticity is described by ellipticity angle θ(λ):
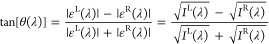
1therefore, the typical measure of ellipticity, θ(λ), is given in degrees. The CD spectrometer measures the differences in intensities for the left (*I*^L^) and right (*I*^R^) circularly polarized light for different wavelengths (λ). In Figure 6a, we present the extinction spectrum of the **TMB** single crystal measured at normal light incidence, i.e., along the chiral *c* axis at 295 K using a JASCO J-1500 CD spectrometer. The fused silica windows of the spectrometer allow measurement of the spectra in the UV–vis range from 220 to 800 nm. The crystal thickness (*d*) was 1.350 mm, and its natural surfaces were not polished. No reference was used for the measurements of crystal CD spectra. The absorption bands (cf. Figure 6a, red line) are located at 274.5, 352.7, 390, 451.2, and 491 nm. The optical transmittance of the investigated crystal is limited by its surfaces that are far from being perfect and scatter incident light, but also, no correction for the reflected light has been applied. The **TMB** crystal shows broad luminescence centered at 580 nm, enabling measurement of the crystal excitation spectrum by detecting emission at a λ_em_ of 580 nm (cf. Figure 6a, black line). Figure 6b presents the circular dichroism spectra of the **TMB** single crystal. Judging from the absorption spectrum of **TMB** in which the strong molecular absorption appears below 250 nm (cf. Figure 6a), one can suppose that the strong CD signal at 239 nm is linked with organic cations ((NH_3_CH_2_CH_2_)_3_NH)^4+^ situated in the structure around the 3-fold chiral axis in the symmetry-related positions. However, the organic molecules themselves are not chiral. It is known from the literature^50−63^ that measurement of the CD in chiral crystals or metamaterials is not as straightforward as in liquid samples containing chiral molecules. Therefore, additional measurements must be performed. First, we checked whether the sign of the circular dichroism is inverted for the crystal with opposite handedness. This was achieved by positioning the **TMB** crystal in a holder, allowing its rotation by 180° (flipping) around the vertical axis to the *c* axis, and next performing CD measurements (Figure 6b). The strongest CD signal changed for the band at 239 nm from positive with θ(239 nm) = 1369 mdeg (red curve) to negative with θ(239 nm) = −1331 mdeg (blue curve). The difference of ∼2.8% in the absolute values of the maximum CD signals for the same band can be linked with the possible asymmetry in crystal mounting as the CD values are sensitive to the rotation of the input crystal face with respect to the **k** vector of incoming light. Other energy bands (274.5 and 352.7 nm) show CD ellipticity θ(λ) at the level of ≤200 mdeg.

**Figure 6:**
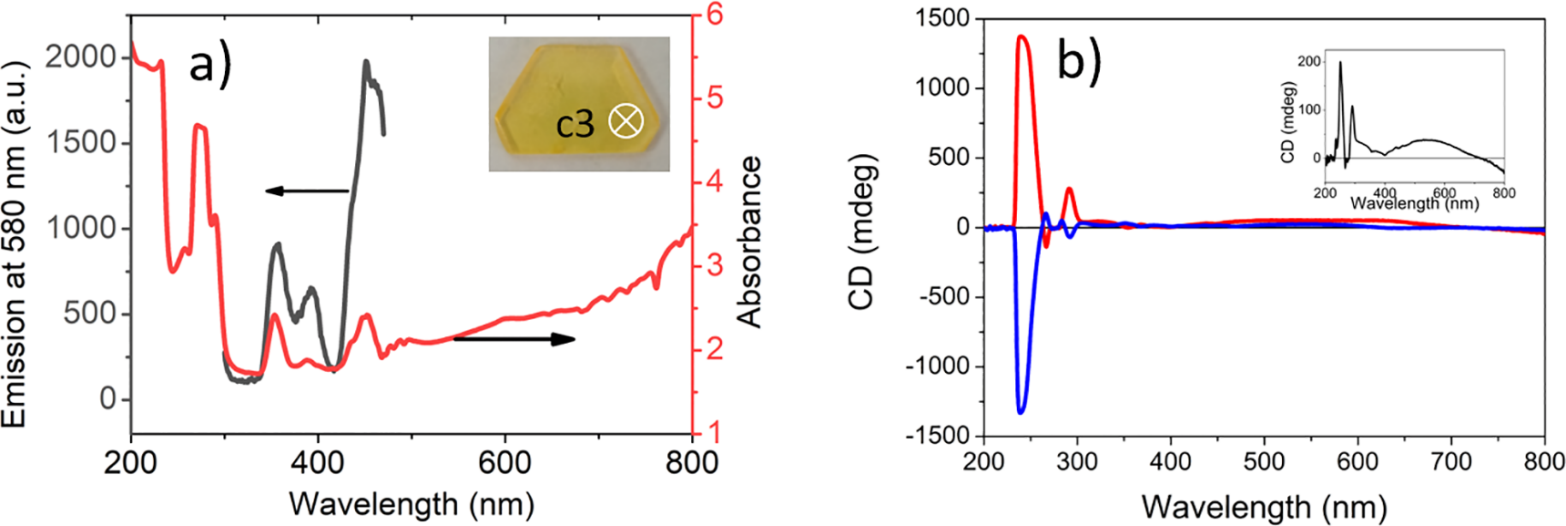
(a) Extinction spectrum (red curve) taken for the **TMB** single crystal with a thickness (*d*) of 1.350 mm along the *c* axis. In the inset, a single crystal is shown with the position of the spectrometer beam incidence marked. The excitation spectrum of the **TMB** crystal (black curve) was measured by recording luminescence emission at 580 nm. (b) Circular dichroism spectra were recorded for the two opposite sides of the **TMB** crystal at 295 K (front side, red curve; rear side, blue curve). The inset shows the average of the two CD spectra.

The large value of the CD signal at 239 nm and calculated from this the anisotropy factor (*g*_CD_ ≈ 0.147)^64^ coming supposedly from the achiral molecules in a macroscopically chiral crystal requires explanation. Recent studies^51,56−58^ have shown that strong CD signals (with macroscopic anisotropy *g*_CD_ factors of ≤0.2) with a marked dependence on the light propagation direction were observed in several organic thin films.^57,58^ In some cases, along with strong CD signals, a complete inversion of the CD spectrum upon flipping of the samples was observed.^51^ The optoelectronic behavior with chiroptical interactions that is antisymmetric with respect to the propagation direction of photons was attributed to an “apparent CD”, and its origin is different from a molecular “genuine CD”, e.g., observed for chiral molecules in solution. According to several earlier publications,^58−60^ the “apparent CD” results from the optical interference of a sample’s linear birefringence (LB) and linear dichroism (LD) rather than excitonic effects^56,57^ but also may be partially attributed to the light interference in the CD apparatus used. The origin of the “apparent dichroism” has been theoretically analyzed by Salij et al.,^58^ who proposed a microscopic theory that includes linear dichroism and linear birefringence interaction in organic thin films using a combination of Mueller calculus and a Lorentz oscillator model. It has been shown that the LD–LB signal may exhibit antisymmetric behavior, i.e., changes in sign upon inversion of the irradiation direction. The genuine chirality together with LD–LB effects can lead to mutual amplification or cancelation. In this work, we have tried to remove the LD–LB antisymmetric contribution by plotting an algebraic average of CD signals obtained for a crystal illuminated from its front and rear face. The result is shown in the inset of Figure 6b. The remnant CD signal is, in this case, much smaller and positive. It must be mentioned that the linear birefringence in the plane perfectly perpendicular to the *c* axis of the **TMB** crystal does not exist because in this plane there is only single ordinary refractive index *n*_o_ = *n*_*xx*_ = *n*_*yy*_. It must be mentioned that “apparent CD” is dependent on the sample thickness and crystal orientation with respect to the light incidence direction. Tilting off the crystal by 30° in a CD spectrometer caused a significant decrease in the “apparent CD” (see Figure S3 and chapter 1.4.1 of the Supporting Information).

The results of CD measurements show that the **TMB** single crystal exhibits circular polarization-dependent absorption. The presence of a large CD signal mostly in the UV range allows its assignment to the electronic state transition within the organic molecules stacked around the 3-fold *c* axis and not to the absorption linked with the inorganic parts of the crystal, i.e., [MnBr_5_]^3–^ trigonal bipyramids. The measurements presented here allowed us to find the sizable “apparent CD” signal due to antisymmetric chiral light–matter interaction. Such crystals, as mentioned by Salij et al.,^58^ may find application in quantum transduction between photonic spin states and the degrees of freedom in matter.

We attempted to determine whether the photoluminescence shows features of circularly polarized light. The **TMB** crystal was excited using linearly polarized light coming from a 473 nm wavelength laser source along the *c* axis. Polarization rotation by 360° by using a dedicated half-wave retardation plate did not result in any noticeable changes in the luminescence intensity. However, we cannot rule out the possibility that the emerging luminescence light might be partially circularly polarized.

In chiral crystals, second-order nonlinear optical effects like second harmonic generation of light or the Pockels effect are allowed for symmetry reasons. We started with the standard Kurtz–Perry^65^ powder method to determine whether SHG can be measured in **TMB** powder. The SHG signal at a λ of 532.15 nm was detected after the sample was analyzed with an optical fiber (Q-Wave) spectrophotometer. The waveguide entrance was situated at an angle of 10° with respect to the IR excitation (1064 nm, 10 ns, repetition of 10 Hz) beam direction. The results described in detail in chapter 1.4.2 of the Supporting Information and Figures S4–S6 were encouraging, allowing for preliminary evaluation of an effective nonlinear coefficient of **TMB** (⟨*d*_TMB_^eff^⟩ ≈ 0.167 pm/V). Then we repeat the SHG measurements for the **TMB** single crystal as a function of the incident energy density for comparison with the results obtained for powder. The results of these measurements are shown in Figure 7. The slope of  versus input light energy density *I*^input^ amounts to 3.43 ± 0.07 arbitrary units J^–1^ cm^–2^, which is larger than that measured for **TMB** powder by a factor of 1.94 ± 0.09. In powder, there are crystallites that are smaller than coherence length *l*_c_, and there is more light scattering that decreases the SHG signal. Upon comparison of slopes obtained for the **TMB** crystal and KDP, the crude estimation of the nonlinear coefficient for **TMB** amounted to 0.27 ± 0.03 pm/V (*d*_11_). The largest SHG coefficients for a standard KDP crytstal are as follows: *d*_36_ = 0.46 pm/V, and *d*_14_ = 0.43 pm/V at 1064 nm.^66^ Note that in this configuration the phase matching condition cannot be realized as irradiation by the fundamental wave is along the crystal optical axis and the beam is not focused. The laser damage threshold for the **TMB** crystal exceeds 250 MW/cm^2^, which is rarely met for organic–inorganic materials.

**Figure 7:**
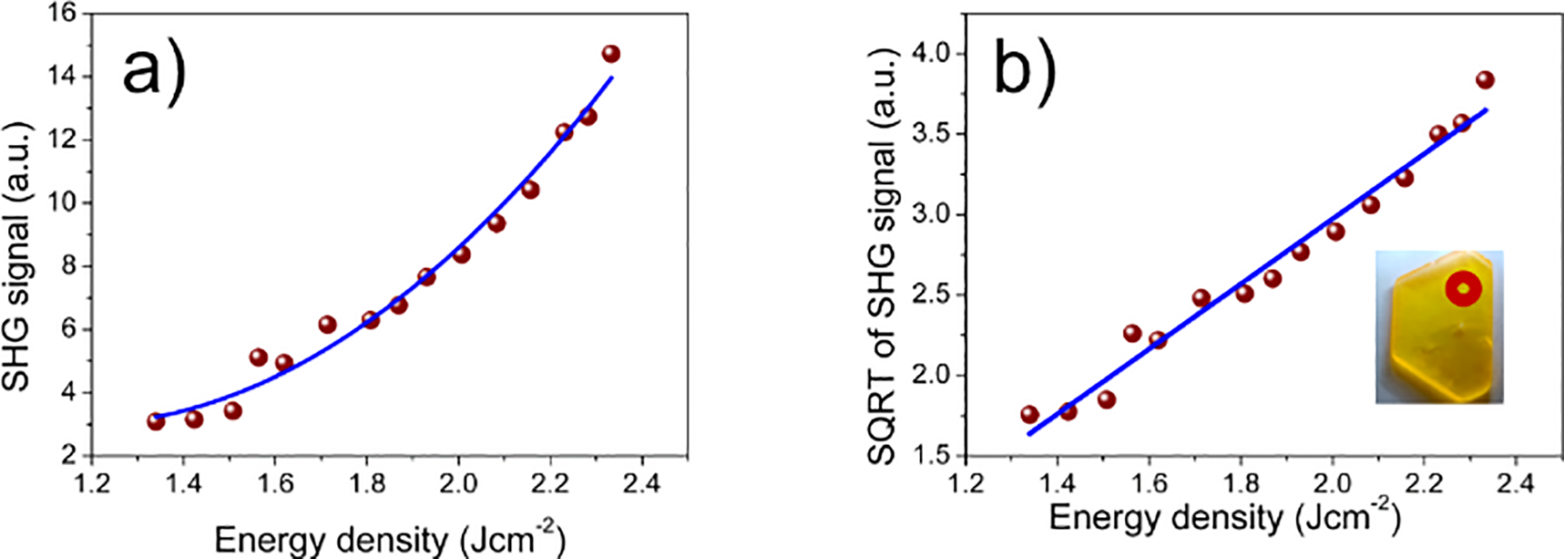
Results of SHG measurements in the single crystal of the **TMB** compound. (a) SHG signal intensity at 532.15 nm vs fundamental beam energy density. (b) Square root of the SHG signal. The slope of a linear fit (blue line) is proportional to the *d*_11_ nonlinear coefficient of the studied compound. The slope of  versus *I*^input^ amounts to 3.43 ± 0.07 (standard error) (arbitrary units) J^–1^ cm^–2^. The inset of panels b shows the position and size of the excitation beam in the **TMB** crystal.

The relatively low value of nonlinear optical tensor coefficients in **TMB** can be rationalized by crystallographic structure analysis. Organic molecules have demonstrated their unique properties for the nonlinear optics due to large values of their first hyperpolarizabilities β_*ijk*_, mostly due to delocalized cloud of π electrons (e.g., “push–pull” or D−π–A architecture systems). In the studied compound, the organic H_4_*tren* cation does not contain any extended π electronic system. It rather represents the nondipolar molecule known as the octupolar one. All dipolar-like quantities, i.e., dipole moment μ_0_ and the vector part of β_*iii*_, vanish for a purely octupolar molecule, and only the symmetry-allowed octupolar components can contribute to second-order NLO effects.^67^ Therefore, the second-order NLO effects are not large. The contribution from the inorganic part could arise from the field-induced asymmetry of trigonal–bipyramidal [MnBr_5_]^3–^ groups. However, without detailed quantum chemical calculations, it is difficult to judge how large their contribution to SHG susceptibilities χ^(2)^ can be.^68^ The role of the N–H···Br hydrogen bonds linking [MnBr_5_]^3–^ anions with cations could not be prevailing due to the weakness of these bonds (see Table S3).

Below, we report a study of a nonlinear chiroptical phenomenon using the technique of a second harmonic generation of light (SHG), and a scheme of the experimental setup is illustrated in Figure S5c. Nonlinear chiroptics has attracted a great deal of attention, especially in the field of chiral metamaterials and plasmonics,^52−55,61−63,69,70^ while it was relatively rarely observed for single crystals.^69−73^ Fu et al.^71^ were the first who reported the SHG-CD effect in a bulk chiral (*P*2_1_2_1_2_1_) single crystal belonging to the organic–inorganic hybrid perovskite family, and we report a similar effect in another chiral (*R*32) bulk organic–inorganic hybrid single crystal of **TMB**. Fortunately, in naturally grown **TMB**, a single crystal dominates a platelet shape with the 3_1_ axis being perpendicular to its largest face. Importantly, for excitation laser light along the *c* axis, SHG can be observed with acceptable efficiency despite the fact that the phase matching of the first and second type cannot be satisfied. Advantageously, in this geometry no beam walk-off is expected, which is crucial for the precision of the optical measurements. Using excitation by circularly left- and right-polarized laser light with the **k** vector along the chiral axis (see the inset of Figure 6a), the SHG circular difference (SHG-CD) can be measured. For polarimetric SHG measurements in a single crystal of **TMB**, a half-wave retardation plate (HWP) or a quarter-wave retardation plate for measurements of SHG-CD was mounted on an automatic rotation stage to control the laser light polarization states. The **TMB** crystal sample was mounted in a holder with its *c* axis along the laser beam **k** vector direction. No analyzers after the sample were used. The case of polarimetric SHG measurement in the **TMB** crystal with excitation by a linearly polarized wave has been modeled and measured as described in chapter 1.4.3 of the Supporting Information (see eqs 5–9 and Figure S7). However, to measure its SHG-CD effect, one needs to use a quarter-wave retardation plate (QWP) with the possibility of its rotation being controlled from 0° to 360°. For that purpose, we mounted a QWP (λ/4 plate for 1064 nm) on the electronically controlled rotatory holder to obtain all possible elliptical states of excitation light polarization: linear, elliptical, and circular left and right polarizations. The constant rotation of QWP allows one to probe the polar plot *I*_2ω_ response at any incident light polarization and thus also LCP and RCP ones. Formally, anisotropy factor *g*_SHG-CD_ is defined as the normalized difference between SHG intensities emerging from the chiral crystal for excitation with RCP and LCP laser light:
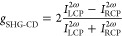
2

Such normalization (eq 2) makes *g*_SHG-CD_ extend between −2 and 2. The results of our measurements of an unpolarized SHG signal with rotating QWP influencing the polarization state of excitation light incident on the **TMB** crystal are shown in Figure 8.

**Figure 8:**
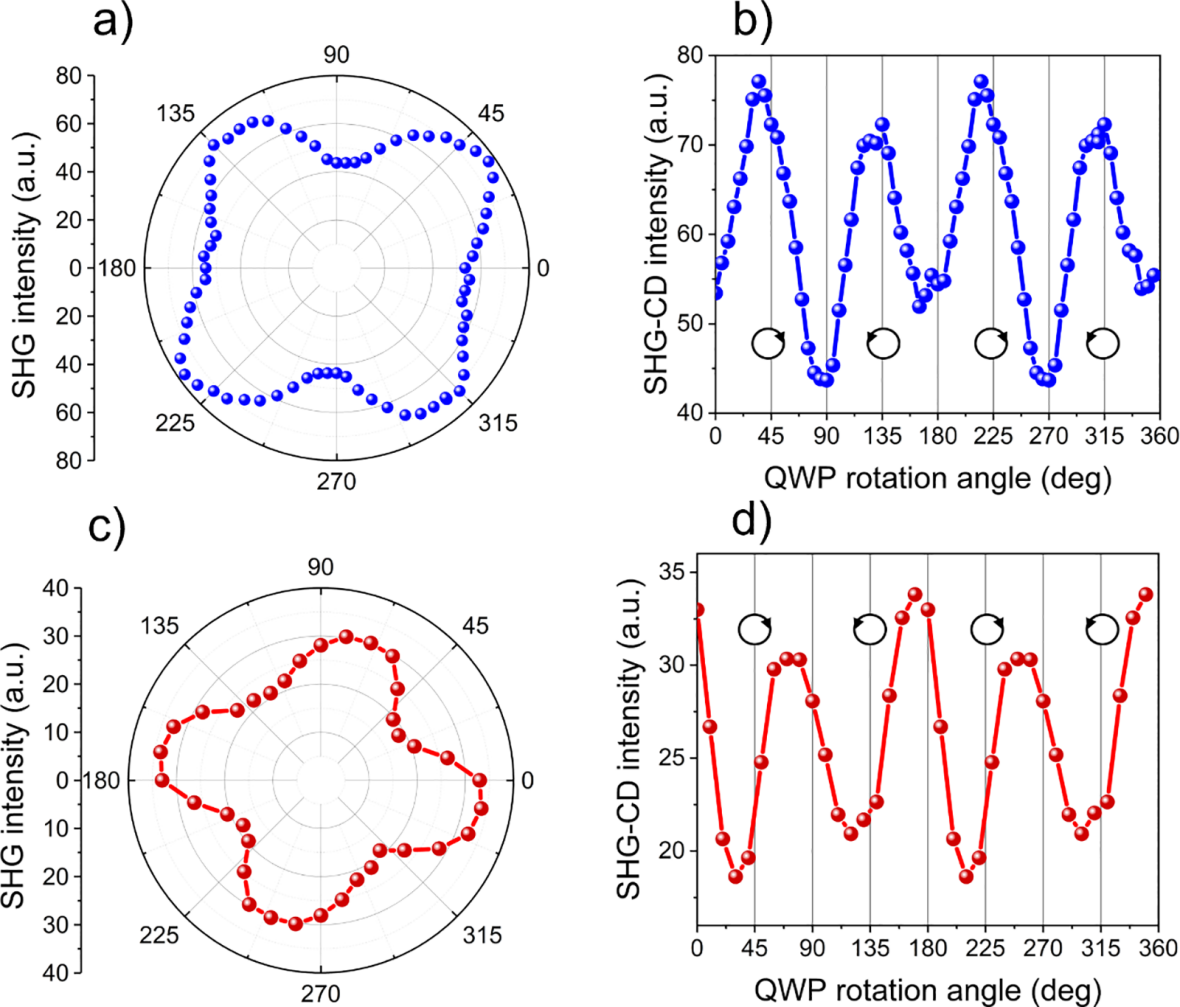
SHG-CD intensities measured for the **TMB** single crystal for two configurations with respect to the *c* polar axis: pointing (a and b) toward the input beam and (c and d) away from it. The λ/4 retardation plate (QWP) was continuously rotated so that the excitation beam polarization was tuned from linear polarization to the left- and right-handed circular polarizations. The output SHG intensity was detected directly without any polarizer.

Plots in Figure 8 demonstrate that the SHG-CD signal modulation reveals no mirror plane with respect to the 180° rotation angle of the λ/4 retardation plate, which is a clear signature of crystal chirality. It is also clear from the plots that the pure LCP and RCP input polarizations do not correspond to the lowest or highest SHG output intensity. Then the simple use of a definition of anisotropy given by eq 2 results in a *g*_SHG-CD_ of 0.062 and might not be sufficient to properly measure the chiral input in this case. To understand the nonlinear chiroptical effects, one needs to go beyond the usual dipolar approximation, which is sufficient to describe NLO phenomena in noncentrosymmetric, nonmagnetic crystals. For chiral crystals, one needs to include magnetic–dipole interactions expressed by the effective χ^(2)eem^ and χ^(2)mee^ tensors:^74^
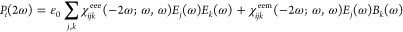
3
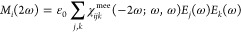
4where *B⃗*(ω) is the magnetic induction field and *M⃗*(2ω) is a nonlinear magnetization.^74^ Both nonlinear polarizations and nonlinear magnetization act as sources for the second harmonic field and can increase or decrease each other depending on the intrinsic chirality of the material. The symmetry properties of nonlinear tensors, including magnetic interactions, are different from those of the electric dipole tensors. A detailed analysis of the expected SHG-CD from a perfect **TMB** crystal under irradiation with linearly polarized laser light transmitted through a rotating QWP is shown in chapter 1.5 of the Supporting Information. The analysis is considerably simplified by treating polarizations involving electric–magnetic contributions as scalars Δχ^chiral^ added to the rigorous tensorial dielectric dipole treatment. The magnitudes of the chiral contributions have been included to properly fit the experimental findings of the SHG-CD experiment for the front and rear faces of the **TMB** crystal.

In Figure 9, we present a summary of the three simulated cases of the SHG-CD response and measurements of the **TMB** single crystal irradiated with infrared laser light with rotated QWP and light incident along the *c* axis on two different faces of the **TMB** crystal.

**Figure 9:**
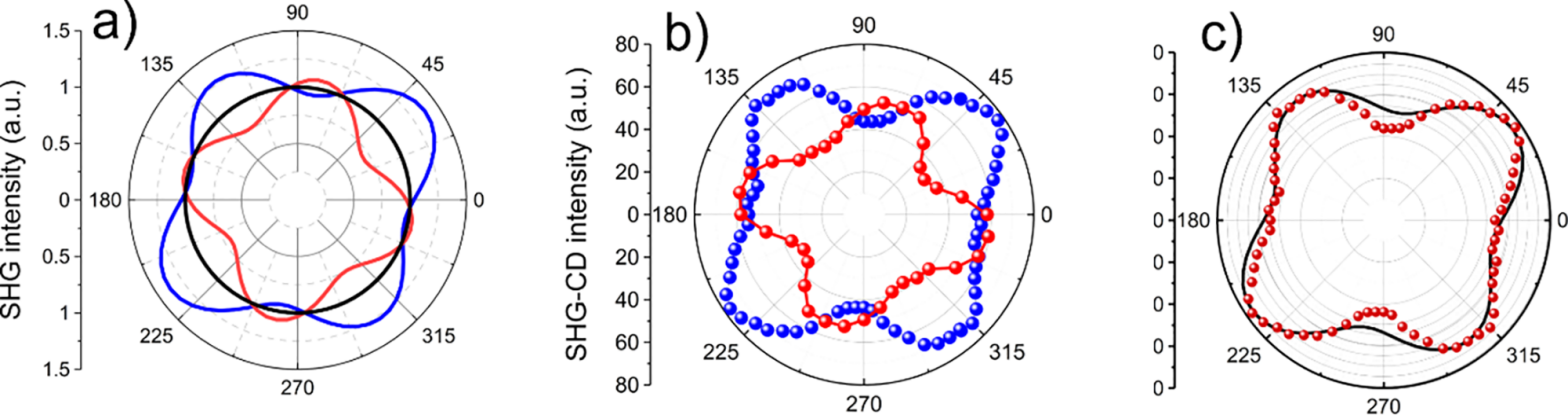
(a) Summary of the three simulations of SHG intensity as a function of the HWP and QWP rotation angle: rotation of linear polarization of excitation beam (black line) and induction of linear, elliptical, and circular polarization (red line or blue line) depending on the crystal polar axis sign. (b) Polar plot of SHG-CD measurements corresponding to panels a and c. (c) Fit of the theoretical function to the measured SHG intensity for the QWP rotation.

A close look at the polar plots shows that the minima and maxima of the SHG-CD signal do not perfectly coincide with the positions of the left and right circular polarizations. This apparent discrepancy originates from the superposition of nonchiral SHG and chiral SHG contributions related to magnetic dipole interactions described in eqs 3 and 4. From the results of the numerical analysis presented in chapter 1.5 of the Supporting Information and Figures S8 and S9, it is evident that the definition of *g*_SHG-CD_ (see eq 2) in the case of a chiral crystal with nonchiral molecules is not straightforward. Only precise measurements and data fitting according to the numerical analysis allow us to estimate the contribution of chiroptic effects properly for a given crystal symmetry, its cut, and the geometry of the experiment.

The numerical fit shown in Figure 9c is not perfect, as our theoretical approach treated an unknown chiral contribution in the simplest possible way by ignoring tensorial susceptibility contributions related to magnetism. Despite that, the agreement between experimental and theoretical predictions is satisfactory. In the measured **TMB** crystal, the relative nonlinear chiroptic contribution is estimated to be 0.25, while that measured according to the definition (see eq 2) amounts to merely 0.062. The **TMB** single crystal shows a significant ability to distinguish between left- and right-handed circular polarizations in the NIR spectral region. Our preliminary results demonstrate its high potential in nonlinear optical, chiroptic, and photonic applications.

According to Figure 1, the **TMB** crystal shows luminescent properties as a characteristic yellow-orange color under UV lamp excitation. The emission spectrum shows a broad band with a maximum at ∼600 nm upon excitation at 450 nm (full width at half-maximum of 2353 cm^–1^), originating from the Mn(II) ion due to its emission from the ^4^T_1_(G) → ^6^A_1_ transition, as reported previously by Mei et al.^26^ Manganese’s electron configuration in the neutral atomic state is [Ar] 3d^5^4s^2^, and the Mn^2+^ ion valence shell is filled with five unpaired electrons. As described elsewhere, the free Mn(II) ion shows a low-intensity spin-forbidden transition (in both absorption and emission).^75^ Analysis of the crystal field splitting is feasible due to crystal field theory. In the case of the examined ion, Tanabe–Sugano diagrams are used to analyze the crystal field interaction, including band gaps between the split energy levels.^76^ The Tanabe–Sugano diagram proves that the emission properties of the Mn(II) ion can be changed relatively easily by changing the coordination environment.

To date, the emission of the Mn(II) ion has been extensively studied and described in tetrahedral and octahedral geometries.^19^ In both given coordinations, the spin-forbidden d–d transition (^4^T_1_ → ^6^A_1_) is visible. It has been shown that it is possible to design the organic (cationic) and inorganic (anionic) parts to obtain different emission colors using manganese compounds. Chen et al.^77^ showed that changing the amount of one of the substrates makes it possible to obtain green or red emission depending on the coordination environment. Despite studies linking the coordination environment of the Mn(II) ion with its luminescent properties, relatively little attention has been paid to compounds in which manganese adopts trigonal–bipyramidal coordination.^26,31^ To more deeply investigate these properties, we measured the emission and excitation spectra of **TMB** powder in a wide temperature range of 20–500 K. At any measurement temperature, we also monitored the associated photoluminescence spectra. The excitation spectrum in the range of 250–500 nm consists of three groups of bands (cf. Figure 10). In the deep UV range, a group of three well-resolved bands is located between 250 and 300 nm; the next group of three bands is located between 340 and 400 nm, with a less intense band at 393 nm. These transitions can be correlated with the ^6^A_1_ → ^4^E_g_(D) and ^6^A_1_ → ^4^T_2g_(D) transitions of the Mn(II) ion. In the visible range, the excitation bands are located between ∼420 and ∼520 nm, corresponding to the ^6^A_1_ → ^4^T_2g_(G) and ^6^A_1_ → ^4^T_1g_(G) transitions, respectively. We can observe the temperature evolution of three distinct groups of excitation bands (see Figure 10), noticing that the luminescence intensity increases 5-fold with an increase in temperature from 20 to 200 K. A majority of the band’s positions do not change except the lowest-energy one, which moves from 500 nm at 20 K to 492 nm at 200 K (see the inset of Figure 10a). Luminescence quenching behavior starts when the sample is heated from room temperature to 500 K, which is visible in Figure 10b. It was shown earlier for manganese-doped compounds that the red emission band correlated with Mn^2+^–Mn^2+^ dimers is fully quenched at ∼463 K.^78^ In the case of the **TMB** compound, luminescence is still visible even above 500 K. At temperatures increasing from room temperature, we observed a shift in the emission intensity maximum toward lower wavelengths like the known in literature reports for Mn(II) doping.^79,80^

**Figure 10:**
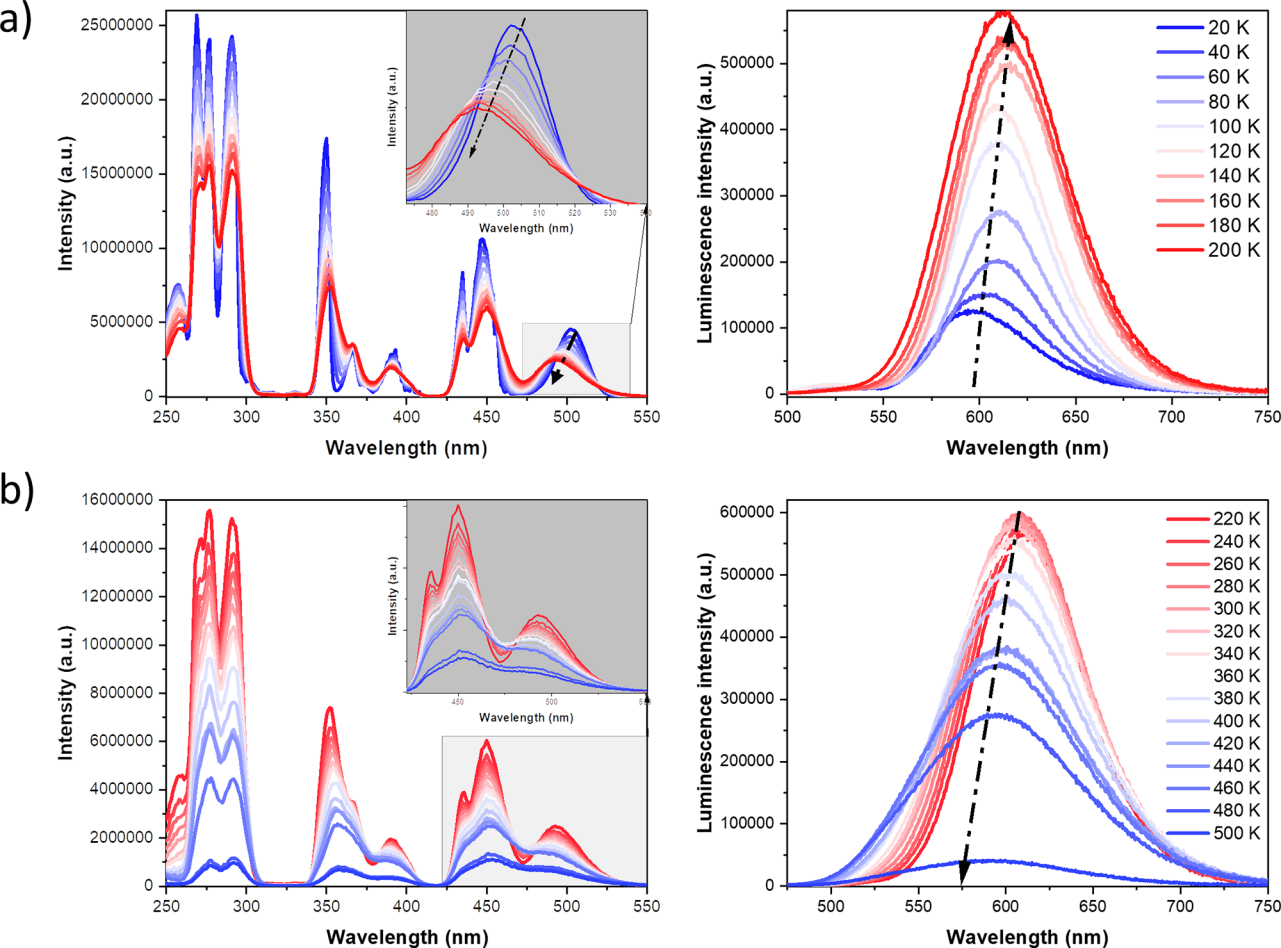
**TMB** powder excitation and emission spectra measured in different temperature ranges: (a) 20–200 and (b) 220–500 K. **TMB** powder emission spectra were excited at 360 nm. Excitation spectra were recorded at 600 nm.

The excitation and absorption spectra recorded for the **TMB** crystal and its powder remain well correlated (see chapter 1.6 of the Supporting Information). All of the optical absorption transitions for Mn^2+^ observed in excitation spectra are forbidden by both parity and spin rules.^75,81^ A characteristic feature of the Mn^2+^ excitation spectrum is the varying spectral width of the absorption transitions. The effect is also predicted by Tanabe–Sugano diagrams,^76^ as a result of the strength of the crystal field changes during atomic vibrations and of temperature changes that control the lattice phonon energy. Generally, the intensities of the lowest-energy absorption and excitation bands of Mn^2+^ located in the range of 400–520 nm are low. This causes disadvantages in potential applications of manganese-based materials. However, great effort was put into improving the emission intensity.^82^ In the case of the crystal in this study, it was possible to obtain material with a high photoluminescence quantum yield. According to the TS diagram,^76,81^ this accounts for all of the observed spectral properties. The broad luminescence band arises from varying slopes of the energy levels; the prolonged decay time results from the spin selection rule, and the emission color varies with the host lattice coordination due to its dependence on the crystal field strength effect on the Mn^2+^ ion. In a tetrahedral coordination, where Mn^2+^ experiences a weak crystal field, the emission is typically green. Conversely, in an octahedral coordination, with a stronger crystal field, Mn^2+^ emits an orange to red color.^83^

The photoluminescence quantum yield of the **TMB** compound, in its powder and single-crystal forms, was measured using integrated sphere and calibrated detectors at room temperature. Its value is quite high, i.e., ∼70% reflecting weak interaction of electronic Mn^2+^ excited states with lattice phonons. Note that the PLQY measured by us is significantly larger than that reported previously.^26^ The luminescence lifetimes under excitation at 360 nm, measured for the **TMB** powder and single crystal, are ∼1.5 ms. This long lifetime of the Mn^2+^ excited state is a result of the spin-forbidden d–d transition (^4^T_1_ → ^6^A_1_). The luminescence decay curves and their fits are shown in Figure S10.

In conclusion, we have studied a new example of a hybrid organic–inorganic crystal (**TMB**) of manganese(II) bromide with branched aliphatic amine [tris(2-aminoethyl)amine] cake-like structure. **TMB** in chiral space group *R*32 exhibits several interesting symmetry-related properties, such as the second harmonic of light generation, optical activity, piezoelectricity, and nonlinear chiroptical effects. The advantage is that the large single crystals can easily be obtained from the solution growth method. The anionic substructure of **TMB** consists of a unique Mn^2+^ trigonal bipyramidal arrangement, allowing for sizable second harmonic circular difference SHG-CD. The latter phenomenon was measured and modeled within a framework of not only usual electric second-order susceptibilities χ^(2)eee^ but also magnetic field-dependent susceptibilities χ^(2)eem^ and χ^(2)mee^. Moreover, **TMB** exhibits intense broadband photoluminescence with a maximum at ∼600 nm upon excitation with UV or violet light. Luminescence originates from the Mn(II) ion due to its relaxation from the excited to ground state ^4^T_1_(G) → ^6^A_1_ transition. The measured lifetime of the emission is quite large, reaching values of 1.5 ms at room temperature, and interestingly, it is accompanied by a very high photoluminescence quantum yield of ∼70%. Lead-free **TMB** crystals may be applied in lighting technologies, chiroptics, and other devices. Further studies of this and similar manganese(II) compounds aim to discover magnetism-related properties and tunable luminescence.

## Supporting Information Available

Experimental details (PDF)
Crystal structure of **TMB** at 100 K (CIF)
Check Platon report (PDF)
Transparent Peer Review report available (PDF)

## Supplementary Material

jz4c00709_si_001.pdf

jz4c00709_si_002.cif
