# Supplementary material for: Nonlinear Optical Activity of a Chiral Organic–Inorganic ([(NH3CH2CH2)3NH])2[MnBr5]Br5 Photoluminescent and Piezoelectric Crystal
Source: J Phys Chem Lett. 2024 May 9;15(19):5276–87. doi: 10.1021/acs.jpclett.4c00709 (PMC11103696; doi:10.1021/acs.jpclett.4c00709)
Supplement: Supplementary file 1 — jz4c00709_si_001.pdf [file jz4c00709_si_001.pdf]

Supplementary Materials

Nonlinear Optical Activity of Chiral Organic-Inorganic ($[(NH_3CH_2CH_2)_3NH])_2[MnBr_5]Br_5$ Photoluminescent and Piezoelectric Crystal

Magdalena Rok[a*], Andrzej Miniewicz[b], Maria Zdończyk[a,c], Bartosz Zarychta[d], Julia W. Mikurenda[a], Stanisław Bartkiewicz[b], Monika Wiśniewska-Bełej[b], Joanna Cybińska*[a,c], Anna Piecha-Bisiorek[a]

[a]Faculty of Chemistry, University of Wroclaw, 14 F. Joliot – Curie, 50-383 Wroclaw, Poland.
[b]Institute of Advanced Materials, Faculty of Chemistry, Wroclaw University of Science and Technology, Wybrzeże Wyspiańskiego 27, 50-370 Wroclaw, Poland.
[c]Łukasiewicz Research Network – PORT Polish Center for Technology Development, ul. Stabłowicka 147, 54-066 Wrocław, Poland.
[d]Faculty of Chemistry, University of Opole, Oleska 48, 45-052 Opole, Poland.

Corresponding authors: magdalena.rok@uwr.edu.pl

Table of contents

1. Experimental 3
1.1 Sample preparation and thermal analysis 3
1.2 Crystal structure determination 4
1.2.1 Details of **TMB** crystal structure 4
1.3 Dielectric measurements and details on piezoelectric effect 7
1.3.1 Dielectric spectroscopy 7
1.3.2 Piezoelectric effect 7
1.4 Circular dichroism and second harmonic generation 8
1.4.1 Circular dichroism in **TMB** crystal 8
1.4.2 SHG experiment in **TMB** powder 9
1.4.3 Second-harmonic generation in **TMB** crystal 12
1.5 Nonlinear chiroptic effect: SHG -CD modelling 14
1.6 Photoluminescence properties 16
2. References 18

CAPTIONS OF FIGURES

Figure S1. The X-ray diffraction pattern at 298 K of **TMB** (pink) and calculated from crystal structure (blue). 3
Figure S2. The simultaneous TGA/DTA analyses for **TMB** (sample mass m = 14.046 mg, 5 K·min^{-1}). 4
Figure S3. Signals of CD measured for **TMB** crystal along the ***c***-axis from front face (red curve), rear face (dark curve) and for tilted by 30 degrees crystal face (red curve) with respect to the incident beam direction. The "apparent CD signal" origin is discussed in main manuscript. 8
Figure S4. The optical microscope photograph of **TMB** powder as seen by objective magnifying 20x. The scale bar 100 μm. 10
Figure S5. Results of SHG measurements in powder of **TMB** compound. a) SHG signal intensity at 532.15 nm versus fundamental beam energy density. b) Square root of the SHG signal. The slope of linear fit (blue line) is proportional to the effective nonlinear coefficient of the studied compound <d^{eff}>. Slope of $\sqrt[2]{I^{SHG}}$ versus I^{input} amounts to: 2.02 and standard error of this estimation is 0.09 in (arb. units)/J·cm^{-2}. c) NLO experimental setup with λ/2 and λ/4 wave plates for 1064 nm laser light used for rotation of linear polarization and preparation of all polarization states, respectively. 11
Figure S6. a) Direct SHG signal intensity comparison at pulse energy density E = 2.2 Jcm^{-2} for KDP powder (red curve) and **TMB** powder (black curve). b) Dependencies of $\sqrt[2]{I^{SHG}}$ versus I^{input} for **TMB** and KDP compounds. 11
Figure S7. Theoretical predictions of expected shapes of SHG intensity for **TMB** crystal upon rotation of linear polarization of Nd:YAG excitation beam: a) polar plot, circular shape (black line) of SHG intensity is expected when no polarizer for generated SHG light is used, blue line corresponds to the measurement of $(P_1)^2$ and red line to the $(P_2)^2$ component of SHG intensity. b) The same result as in (a) plotted in a linear plot with respect to HWP rotation angle. c) Experimental result of SHG intensity obtained with full rotation of half-wave plate, i.e., incident linear polarization rotation by 360°, on polar plot the circular shape has been measured. d) SHG intensity versus HWP rotation angle in a linear plot with respect to HWP rotation angle. 14
Figure S8. Methodology of creating the expected shape of SHG-CD in **TMB** single crystal with positive $\Delta\chi^{chiral}$ = 0.032 and χ^{eem} = 0.25. a) function [0.032+0.25 sin(2ϕ)] versus QWP rotation angle ϕ; b) function [0.032+0.25 sin(2ϕ)]^2, with SHG-CD = 0.249; c) polarimetric plot of chiroptic contribution to SHG; d) plots of $P_x(2\omega)$ + 0.5[0.032+0.25 sin(2ϕ)] and $P_y(2\omega)$ + 0.5[0.032+0.25 sin(2ϕ)] ; e) plot of [$P_x(2\omega)$ + 0.5[0.032+0.25 sin(2ϕ)]]^2 +[$P_y(2\omega)$ + 0.5[0.032+0.25 sin(2ϕ)]]^2, here SHG-CD = 0.0306 and f) polarimetric plot of the last function. 15
Figure S9. Methodology of creating the expected shape of SHG-CD in **TMB** single crystal with negative $\Delta\chi^{chiral}$ = – 0.032 and χ^{eem} = – 0.25. a) function – [0.032+0.25 sin(2ϕ)] versus QWP rotation angle ϕ; b) function – [0.032+0.25 sin(2ϕ)]^2, with SHG-CD = 0.249; c) polarimetric plot of chiroptic contribution to SHG; d) plots of $P_x(2\omega)$ – 0.5[0.032+0.25 sin(2ϕ)] and $P_y(2\omega)$ – 0.5[0.032+0.25 sin(2ϕ)] ; e) plot of [$P_x(2\omega)$ – 0.5[0.032+0.25 sin(2ϕ)]]^2 +[$P_y(2\omega)$ – 0.5[0.032+0.25 sin(2ϕ)]]^2, here SHG-CD = 0.0312 and f) polarimetric plot of the last function. 16
Figure S10. Luminescence decay times measured for a) powder sample; b) crystal sample of **TMB**.. 17

CAPTIONS OF TABLES

Table S1. Experimental details for **TMB**. 5
Table S2. Geometric parameters (Å, °) for **TMB**. 6
Table S3. Hydrogen-bond geometry (Å, º) for **TMB**. 6

1. Experimental

1.1 Sample preparation and thermal analysis

1.96 g of $MnBr_2{\cdot}4H_2O$ (Sigma-Aldrich, 98%, 6.8 mmol) was dissolved in deionized water, then concentrated hydrobromic acid (4 ml, 10 mmol 57% Sigma Aldrich) was added dropwise to the solution. Next, tris(2-aminoethyl)amine (Sigma-Aldrich, 96%, 1g, 6.8 mmol) was added in part to the solution placed in the ice bath. By slow evaporation at room temperature, yellow crystals of $(C_6N_4H_{22})_2[MnBr_5]Br_5$, **TMB** were obtained after two weeks. The composition of the compound was confirmed by elemental analysis to be C: 12.62% (*theor.* 12.48%), N: 9.72% (*theor.* 9.71), H 3.96% (*theor*. 3.84%). A powder X-ray diffraction verified the phase purity (see Figure S1). XRD was recorded in the range 2θ = 5–80° with the step 2θ = 0.0241 and 1 s counting time using a D8 Advance X-ray Diffractometer from Bruker. The Ni-filtered Cu Ka1 radiation (λ = 1.540596 Å) from a Cu X-ray tube was applied.

The thermal stability of the crystal was checked by thermogravimetric analysis (TGA) and the differential scanning calorimetry (DSC) (Mettler Toledo DSC3+) in the temperature range of 300−900 K with a ramp rate of 5 $K{\cdot}min^{-1}$ (Figure S2). The scans were performed in flowing nitrogen (flow rate: 1 $dm^3{\cdot}h^{-1}$).

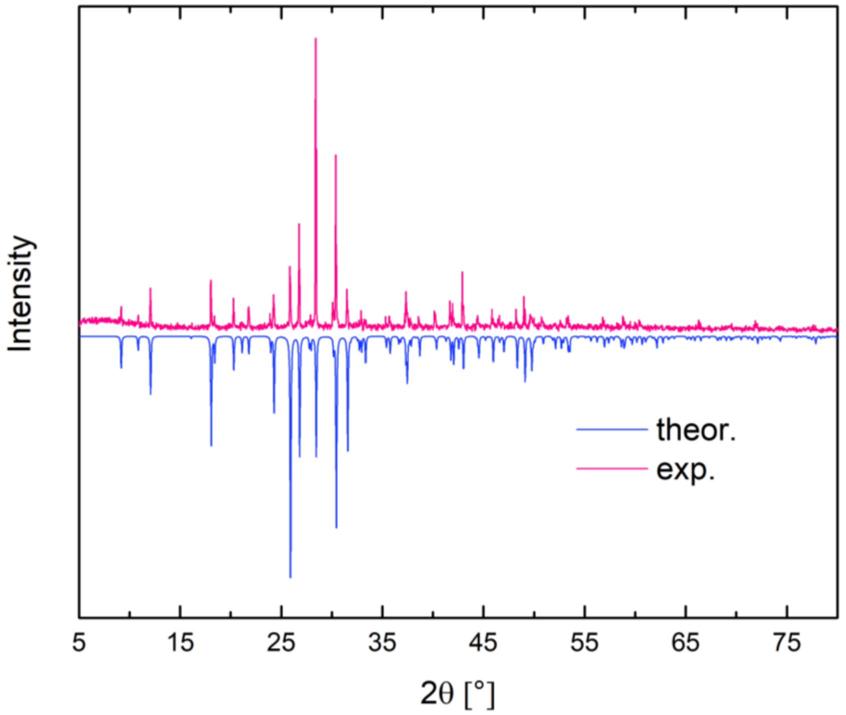

Figure S1. The X-ray diffraction pattern at 298 K of **TMB** (pink) and calculated from crystal structure (blue).

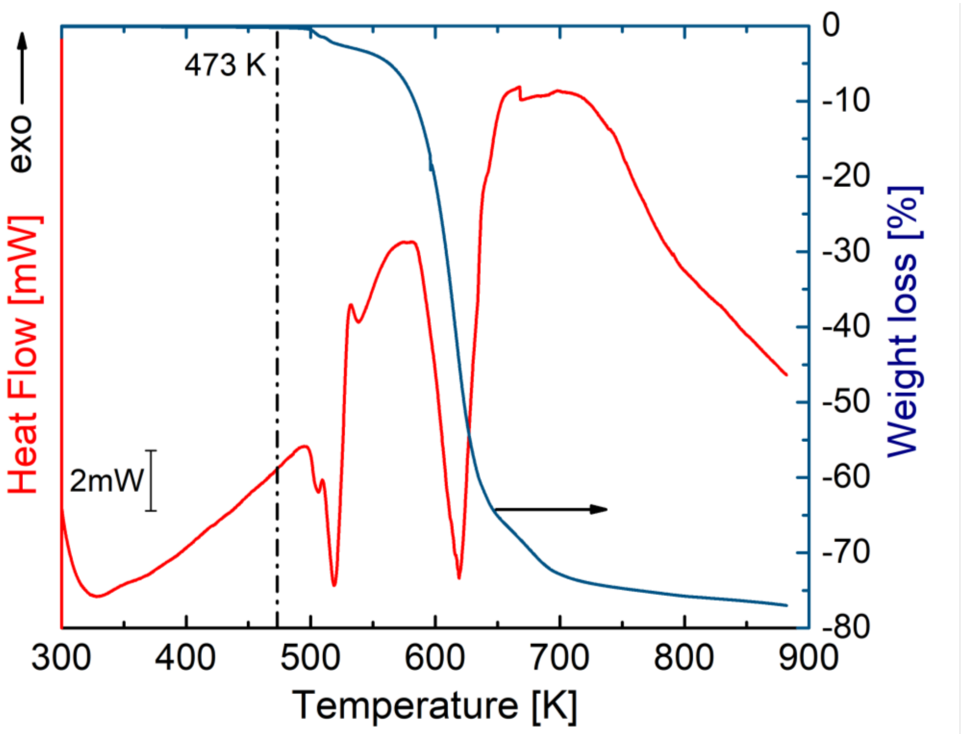

Figure S2. The simultaneous TGA/DTA analyses for **TMB** (sample mass m = 14.046 mg, 5 K·min^{-1}).

1.2 Crystal structure determination

1.2.1 Details of **TMB** crystal structure

The single crystal of the **TMB** was mounted on a Rigaku XtaLAB Synergy R, DW system, HyPix-Arc 150 (graphite monochromatic, Mo*K*α radiation, λ = 0.71073 Å). The measurements have been done at 100.0(1) K. The corrections to the Lorentz and polarization factors were applied to the reflection intensities.[1] The structures were solved by direct methods applying SHELX program package.[2] Graphics made with Mercury 2022.3.[3] The hydrogen atoms were determined from the geometric concepts for C-H and from a difference Fourier map for all N-H. The C-H hydrogen atoms were refined in a riding model with isotropic temperature factors of 1.2 times the U_{eq} value of the parent atom while N-H in an independent atom model with isotropic temperature factors of 1.5 times the U_{eq} value of the parent atom. All non-hydrogen atoms were located from difference Fourier synthesis and refined by least squares method in the full-matrix anisotropic approximation. The crystallographic data for the compound and details of X-ray experiment are collected in the Table S1. The crystallographic data have been deposited with the Cambridge Crystallographic Data Centre no. 2259681 (12 Union Road, Cambridge CB2 1EZ, UK (Fax, 44-(1223)336-033, E-mail deposit@ccdc.cam.ac.uk).

Table S1. Experimental details for **TMB**.

Crystal data	
Chemical formula	$(C_6N_4H_{22})_2[MnBr_5]Br_5$ (**TMB**)
M_r	1154.59
Crystal system, space group	Trigonal, *R*32:*H*
Temperature (K)	100
a, *c* (Å)	9.8125 (2), 28.643 (1)
V ($Å^3$)	2388.41 (13)
Z	3
Radiation type	Mo *K*α
μ (mm^{-1})	12.98
Crystal size (mm)	0.05 × 0.05 × 0.05
Data collection	
Diffractometer	XtaLAB Synergy R, DW system, HyPix-Arc 150
Absorption correction	Multi-scan<br>*CrysAlis PRO* 1.171.41.93a (Rigaku Oxford Diffraction, 2020) Empirical absorption correction using spherical harmonics, implemented in SCALE3 ABSPACK scaling algorithm.
T_{min}, T_{max}	0.772, 1.000
No. of measured, independent and observed [$I > 2\sigma(I)$] reflections	4178, 988, 944
R_{int}	0.026
$(\sin \theta/\lambda)_{max}$ ($Å^{-1}$)	0.616
Refinement	
$R[F^2 > 2\sigma(F^2)]$, $wR(F^2)$, S	0.014, 0.034, 1.05
No. of reflections	988
No. of parameters	50
H-atom treatment	H atoms treated by a mixture of independent and constrained refinement
$\Delta\rho_{max}$, $\Delta\rho_{min}$ (e $Å^{-3}$)	0.73, −0.35
Absolute structure	Flack x determined using 335 quotients [(I+)-(I-)]/[(I+)+(I-)] (Parsons, Flack and Wagner, Acta Cryst. B69 (2013) 249-259).
Absolute structure parameter	0.016 (14)

Computer programs: CrysAlis CCD, Oxford Diffraction, 2007, CrysAlis RED, SHELXL2014/7 (Sheldrick, 2014), Mercury 2020.3.0 (Macrae et al., 2020).

Table S2. Geometric parameters (Å,°) for **TMB**.

Bonds			
Mn1—Br1^{i}	2.4842 (4)	C2—C3	1.519 (5)
Mn1—Br1	2.4842 (5)	C2—H2A	0.9900
Mn1—Br1^{ii}	2.4842 (4)	C2—H2B	0.9900
Mn1—Br2^{iii}	2.9071 (5)	C3—N4	1.486 (4)
Mn1—Br2	2.9072 (5)	C3—3A	0.9900
N1—C2^{iv}	1.514 (3)	C3—H3B	0.9900
N1—C2^{v}	1.514 (3)	N4—H4A	0.8296
N1—C2	1.514 (3)	N4—H4B	0.8613
N1—H1	1.00 (6)	N4—H4C	0.9559
Angles			
Br1^{i}—Mn1—Br1	120.0	N1—C2—H2A	109.7
Br1^{i}—Mn1—Br1^{ii}	120.0	C3—C2—H2A	109.7
Br1—Mn1—Br1^{ii}	120.0	N1—C2—H2B	109.7
Br1^{i}—Mn1—Br2^{iii}	90.0	C3—C2—H2B	109.7
Br1—Mn1—Br2^{iii}	90.000 (1)	H2A—C2—H2B	108.2
Br1^{ii}—Mn1—Br2^{iii}	90.002 (1)	N4—C3—C2	109.3 (3)
Br1^{i}—Mn1—Br2	90.0	N4—C3—3A	109.8
Br1—Mn1—Br2	90.0	C2—C3—3A	109.8
Br1^{ii}—Mn1—Br2	90.0	N4—C3—H3B	109.8
Br2^{iii}—Mn1—Br2	180.0	C2—C3—H3B	109.8
C2^{iv}—N1—C2^{v}	110.98 (19)	3A—C3—H3B	108.3
C2^{iv}—N1—C2	110.98 (19)	C3—N4—H4A	111.0
C2^{v}—N1—C2	110.98 (19)	C3—N4—H4B	109.5
C2^{iv}—N1—H1	107.92 (19)	H4A—N4—H4B	113.3
C2^{v}—N1—H1	107.9 (2)	C3—N4—H4C	112.6
C2—N1—H1	107.9 (2)	H4A—N4—H4C	109.2
N1—C2—C3	109.8 (3)	H4B—N4—H4C	100.9
Dihedral Angles			
C2^{iv}—N1—C2—C3	−81.4 (4)	N1—C2—C3—N4	169.5 (2)
C2^{v}—N1—C2—C3	154.7 (3)		

Symmetry codes: (i) −y+1, x−y+1, z; (ii) −x+y, −x+1, z; (iii) y−1/3, x+1/3, −z+4/3; (iv) −y, x−y, z; (v) −x+y, −x, z.

Table S3. Hydrogen-bond geometry (Å, º) for **TMB**

D—H···*A*	*D*—H	H···*A*	*D*···*A*	*D*—H···*A*
N1—H1···Br4	1.00 (6)	2.23 (6)	3.230 (4)	180 (1)
C2—H2*A*···Br3^{i}	0.99	3.01	3.812 (3)	139
C2—H2*B*···Br3^{vi}	0.99	3.07	3.770 (3)	129
C3—3A···Br1^{ii}	0.99	3.01	3.814 (3)	139
C3—3A···Br4	0.99	3.06	3.684 (3)	123
C3—H3*B*···Br1^{vii}	0.99	2.97	3.857 (3)	149

Symmetry codes: (i) −y+1, x−y+1, z; (ii) −x+y, −x+1, z; (vi) −x+y−1, −x, z; (vii) x−1, y−1, z.

1.3 Dielectric measurements and details on piezoelectric effect

1.3.1 Dielectric spectroscopy

The complex dielectric permittivity, $\varepsilon^* = \varepsilon' - i\varepsilon''$, was measured between 100 and 350 K by an Agilent E4980A Precision LCR Meter in the frequency range from 135 Hz to 2 MHz. The overall error for the real and imaginary parts of the complex dielectric permittivity was less than 5% and 10%, respectively. The single crystals were crystallographically oriented along the ***c***-axis and the surfaces of the crystals were coated with silver conductive paint (Electron Microscopy Sciences, 503).

1.3.2 Piezoelectric effect

Piezoelectricity is the ability of electric charge accumulation in non-centrosymmetric solid materials in response to the applied mechanical stress or strain. Stress and strain are related with an electric field *E* via a third order tensor:

$$e_{ijk}^{T} = \left(\frac{\partial D_i}{\partial \epsilon_{jk}}\right)_{E,T} = -\left(\frac{\partial \sigma_{jk}}{\partial E_i}\right)_{\epsilon,T} \qquad \text{(Eq. 1)}$$

where *D*, *E*, ϵ, σ and *T* represent the electric displacement field, the electric field, the strain tensor, the stress tensor and the temperature, respectively. In the literature the piezoelectric strain constant obtained from the experiments is usually denoted as d_{ijk}. These can be readily related to the e_{ijk} constants if the elastic compliances $s_{lmjk}^{E,T}$ of the material are known, then:

$$d_{ijk}^{T} = e_{ilm} s_{lmjk}^{E,T} \qquad \text{(Eq. 2)}$$

where summation over repeated indices is assumed[4]. For the trigonal system and point group 32 form of the piezoelectric tensor is following:[4]

$$\begin{pmatrix} e_{11} & -e_{11} & 0 & e_{14} & 0 & 0 \\ 0 & 0 & 0 & 0 & -e_{14} & -e_{11} \\ 0 & 0 & 0 & 0 & 0 & 0 \end{pmatrix} \qquad \text{(Eq. 3)}$$

where piezoelectric e_{ijk}^{T} tensor has been written in shortened matrix Voigt notation. So in the **TMB** crystal there are only two independent piezoelectric coefficient that need to be measured. From the work of de Jong, et al.[4] and Figure 6 therein, it follows that in the chiral point group 32 the available, reported until now, data point out that the largest values of piezoelectric tensor $\|e_{ijk}^{T}\|$ do not exceed 2.5 C/m^2. In our experiment we observed the reverse piezoelectric effect, the internal generation of a mechanical strain was caused by externally applied *AC* electric field (E_3 || *c*-axis) used for the measurement of complex dielectric response.

Considering the structure-property relationship it is reasonable to assume that the observed

strongest piezoelectric resonance at 133 kHz for **TMB** single crystal is related to the lowest acoustic resonance with acoustic wave propagating within the direction perpendicular to the *c*-axis. Taking into account that the resonance is broad one may suppose that it can represent a mixture of several acoustic waves being close to resonance conditions with crystal edges seen in Figure 1 of the main manuscript. For the inducement of piezoelectricity can be responsible the shearing strain that can deform the triangular base of bipyramids formed by Mn^{2+} and three Br1 atoms, however other charged units may be involved as well. It is well known that the measurement as well as calculations of piezoelectric crystal response is a difficult task and the results reported in literature for the seemingly the same material may differ in the values of piezoelectric coefficients due to material imperfections, defects, domains, etc.

1.4 Circular dichroism and second harmonic generation

1.4.1 Circular dichroism in **TMB** crystal

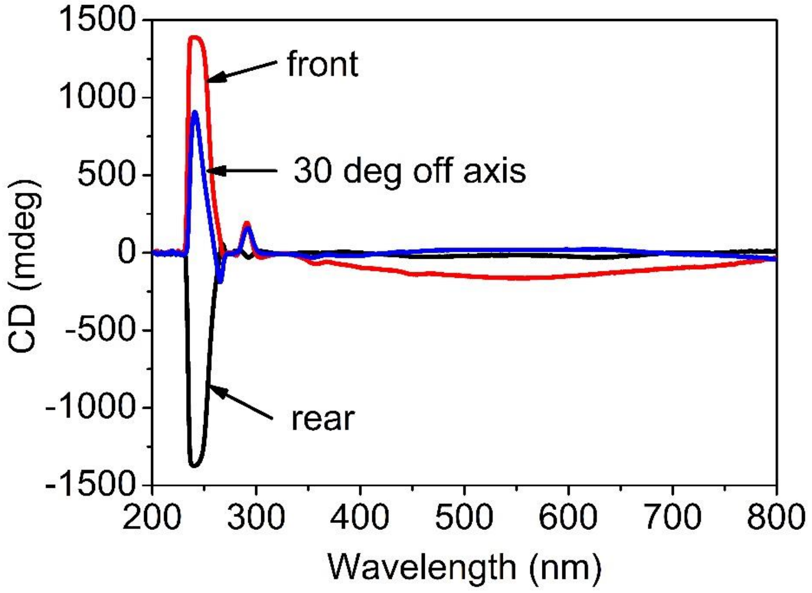

Figure S3. Signals of CD measured for **TMB** crystal along the ***c***-axis from front face (red curve), rear face (dark curve) and for tilted by 30 degrees crystal face (red curve) with respect to the incident beam direction. The "apparent CD signal" origin is discussed in main manuscript.

The measured CD signal is not linked with the chirality of the molecules, because they are not chiral, but comes from the crystal structure chirality with the organic molecules occupying three symmetry related positions around the 3-fold chiral axis. Similar situation is observed in chiral nematics (nematic liquid crystals) in which structural chirality is introduced by addition of a

small amount of chiral dopant. Achiral molecules minimize energy of interaction with chiral molecule that leads to breaking symmetry and appearance of a helicoidal structure. In the described case the pitch p of the helix is controlled by the dopant helical twisting power and its concentration. In **TMB** crystal the pitch is equal to a period of the crystal along the *c*-axis.

1.4.2 SHG experiment in **TMB** powder

In the case of newly synthesized hybrid crystals, when only crystalline powder is available the Kurtz-Perry[5] second harmonic generation (SHG) powder technique is the only choice in preliminary screening the material for interesting second order NLO properties. This technique allows for measurement of an effective nonlinear optical coefficient $< d_{il}^{eff} >$ for a crystalline compound in the form of powder in reference to the standard compound with known NLO tensor coefficients. Its versatility has been proven by hundreds of measurements of different second order NLO materials including oxides, hydroxides, chalcogenides, halides, borates, carbonates, iodates, organic crystals, etc. However, as any simple and versatile technique, Kurtz-Perry technique is not free from the possible errors in evaluation of the values of NLO coefficients for new materials. Kurtz-Perry powder SHG technique does not consider effects of anisotropic light scattering in the SHG process; it can be sensitive for the packing fraction of the powder, sample thickness, forward or backward experiment scattering geometry and even a choice of a reference material. Powder samples are constituted of randomly oriented crystals, frequently of different sizes and shapes, and the total SHG response is the sum of the contributions from all individual particles.[6] The evolution of the SHG signal that arrives to detector is dependent on the length travelled by light inside the individual crystal and on magnitude and dispersion of its refractive indices as well as on scattering process. These factors should always be considered when drawing conclusions from measurements by Kurtz-Perry[5] powder SHG technique.

SHG signal was excited by Q-switched pulse laser (Nd^{3+}:YAG, Surelite II) working at fundamental wavelength of $\lambda = 1.064$ μm and delivering ~10 ns pulses with repetition rate of 10 Hz. The unfocused beam of infrared radiation of 5 mm in diameter was incident on the centre of powder sample. Measurements of SHG intensities in function of incident laser pulse energy were performed in the range of 20 to 400 mJ. The laser output energy was changed using time delay of opening of introduced into the laser cavity Pockels cell with respect to the excitation lamp pulse. Laser power was measured using calibrated energy power meter. A fibre optics spectrometer (Qwave, RGB Photonics) enabled collection of the green ($\lambda = 532$ nm) SHG scattered light via optical fibre mounted at the sample holder and measured the spectrum from

300 to 900 nm. Measurement time was set to 0.05 s and final results were obtained by averaging 100 measurements for a chosen single energy density of fundamental beam. The reference sample KH_2PO4 (KDP powder) was measured exactly the same way. All measurements were performed at room temperature of 295 K. The SHG signal appeared at wavelength 532.15 nm as single narrow peak characterized by the width FWHM = 0.55 nm.

The yellowish in colour powder of **TMB** compound was comprised of irregular shape of crystalline grains within the 50 to 300 μm size range (see Figure S4). Powder was confined in between two microscope glass plates making circle of diameter around 14 mm. A 0.3 mm-thick spacer was inserted between the glass slides to ensure the consistency of the sample thickness, and next sealed with a tape. Sample was inserted and fixed in a special holder equipped also with the optical fibre holder positioned at the angle 30° with respect to the excitation beam incidence direction and the surface normal. In the used forward SHG scattering geometry of an experiment the entrance aperture of the fibre collected only a small portion of the total SHG signal. We prepared also the reference powder sample from KDP crystal characterized by the grain sizes *r* between 80 and 120 μm.

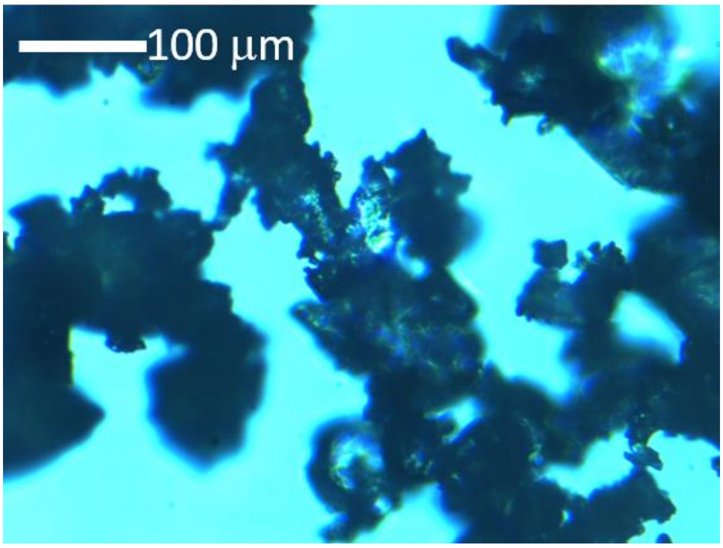

Figure S4. The optical microscope photograph of **TMB** powder as seen by objective magnifying 20x. The scale bar 100 μm.

In Figure S5 we present the measurements of SHG intensity response of **TMB** powder in function of excitation laser pulse energy density. The parabolic dependence of SHG signal with respect to fundamental beam intensity shows expected quadratic behaviour.

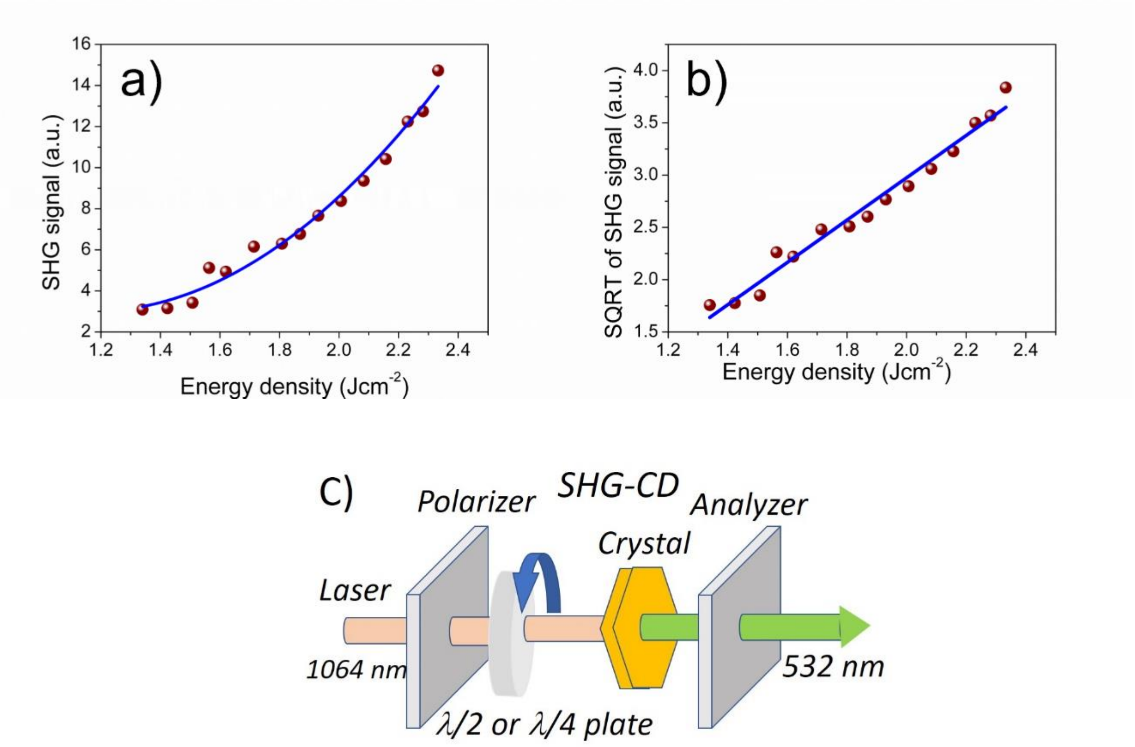

Figure S5. Results of SHG measurements in powder of **TMB** compound. a) SHG signal intensity at 532.15 nm versus fundamental beam energy density. b) Square root of the SHG signal. The slope of linear fit (blue line) is proportional to the effective nonlinear coefficient of the studied compound <*d*^{eff}>. Slope of $\sqrt[2]{I^{SHG}}$ versus I^{input} amounts to: 2.02 and standard error of this estimation is 0.09 in (arb. units)/J·cm^{-2}. c) NLO experimental setup with λ/2 and λ/4 wave plates for 1064 nm laser light used for rotation of linear polarization and preparation of all polarization states, respectively.

In Figure S6a we directly compare SHG signals measured for standard KDP powder with that of **TMB** compound for energy density of fundamental frequency (ω) beam E = 2.2 J·cm^{-2}. In Figure S 6b we compare the dependencies of SHG intensities via $\sqrt[2]{I^{SHG}}$ in studied **TMB** powder and reference powder of KDP versus energy density in exactly the same experimental conditions

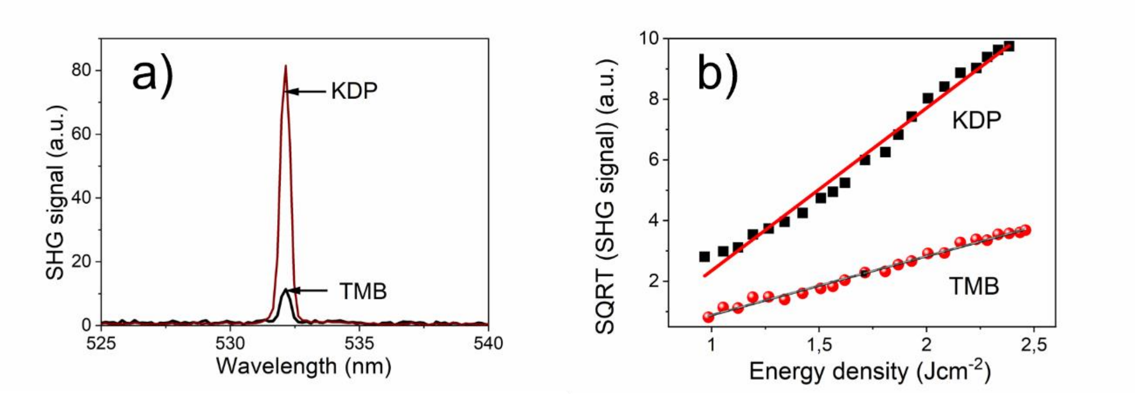

Figure S6. a) Direct SHG signal intensity comparison at pulse energy density E = 2.2 Jcm^{-2} for KDP powder (red curve) and **TMB** powder (black curve). b) Dependencies of $\sqrt[2]{I^{SHG}}$ versus I^{input} for **TMB** and KDP compounds.

The plots $\sqrt[2]{I^{SHG}}$ versus I^{input} allow for more accurate estimation of effective nonlinear coefficient of **TMB** compound. The slope of linear dependence shown for KDP is 5.364 with standard error 0.15 and slope for **TMB** is 1.94 with standard error 0.083. Using the equation:

$$\langle d_{TMB}^{eff} \rangle = \langle d_{KDP}^{eff} \rangle \cdot \left(\frac{I_{TMB(2\omega)}}{I_{KDP(2\omega)}} \right)^{1/2} \quad \text{(Eq. 4)}$$

and the slopes of linear fits one obtains $\frac{\langle d_{KDP}^{eff} \rangle}{\langle d_{TMB}^{eff} \rangle} = 2.67$ what results in $\langle d_{TMB}^{eff} \rangle \approx 0.167$ pm/V assuming for KDP $\langle d_{KDP}^{eff} \rangle \approx 0.43$ pm/V.[7] The relatively small powder SHG response of the studied crystal can be explained by the presence of **TMB** crystal grains with size smaller than the coherence length l_c for SHG process. However, it is worth to mention that the optical damage threshold for **TMB** compound measured at λ = 1064 nm and 10 ns duration laser pulses exceeds 250 MW/cm^2, as no departure of SHG intensity versus laser energy density from the parabolic behaviour has been identified. Such a large value of optical damage threshold is not frequently observed among the family of organic-inorganic hybrid perovskites, thus it is a good prognostic for the possible application of **TMB** crystals in photonics.

1.4.3 Second-harmonic generation in **TMB** crystal

Second harmonic generation is a parametric degenerate sum frequency NLO process in which two photons of energy $\hbar\omega$ annihilate and create a single photon of energy $2\hbar\omega$. Second-order nonlinear polarization in a dipolar approximation is described as:

$$P_i(2\omega) = \varepsilon_0 \sum_{j,k} \chi_{ijk}^{eee}(-2\omega; \omega, \omega) E_j(\omega) E_k(\omega) \quad \text{(Eq. 5)}$$

where $\chi_{ijk}^{eee}(-2\omega; \omega, \omega)$ is an electric-dipole allowed second order susceptibility, $E_j(\omega)$ are electric field amplitudes of the incident light and the indices *i,j,k* refer to the Cartesian coordinates. Susceptibility tensor $\chi_{ijk}^{eee}(-2\omega; \omega, \omega)$ is related to d_{il}-matrix via relation $d_{ijk} = \frac{1}{2}\chi_{ijk}^{(2)}$, where *i,j,k* = 1, 2 or 3, and *l* = 1, …,6. (Note that the symmetry of the piezoelectric tensor (cf. Eq. 3) is identical with that of NLO susceptibility.) The studied **TMB** compound belongs to the trigonal system and *R*32 space group (like SiO_2 or Te crystals). This symmetry, according to the Neumann's principle, together with the Kleinman symmetry conditions reduce the number of nonlinear tensor coefficients to only two independent ones: d_{11} and d_{14}. The nonlinear polarization vector components $P_i(2\omega)$ can be obtained from the matrix equation[8] in the form:

$$\begin{bmatrix} P_1(2\omega) \\ P_2(2\omega) \\ P_3(2\omega) \end{bmatrix} = 2\varepsilon_0 \begin{bmatrix} d_{11} & -d_{11} & 0 & d_{14} & 0 & 0 \\ 0 & 0 & 0 & 0 & -d_{14} & -d_{11} \\ 0 & 0 & 0 & 0 & 0 & 0 \end{bmatrix} \begin{pmatrix} E_1^2 \\ E_2^2 \\ E_3^2 \\ 2E_2E_3 \\ 2E_1E_3 \\ 2E_1E_2 \end{pmatrix} \quad \text{(Eq. 6)}$$

Experimental geometry with an unfocused excitation beam incident along the ***c***-axis of **TMB** crystal defines **k** = (0, 0, k$_3$) and $\boldsymbol{E} = (E_1, E_2, 0)$, then two components of nonlinear polarization can appear:

$$P_1(2\omega) = 2\varepsilon_0(d_{11}E_1^2 - d_{11}E_2^2) \text{ and } P_2(2\omega) = -4\varepsilon_0 d_{11}E_1E_2, \quad \text{(Eq. 7)}$$

in laboratory coordinate frame the **z**-axis corresponds to the crystallographic ***c***-axis (in the notation used in equations 5 to 7: x=1, y=2 and z=3).

In fact, the linear polarization of incident light d_{11} coefficient can be directly measured in this configuration. We used a half-wave retardation plate (HWP) for λ = 1064 nm to rotate the linear polarization of the laser beam by 360°. The SHG signal for any azimuthal linear polarization of the excitation beam should result in the same intensity $I_{2\omega}$ under the condition that there is no linear polarizer inserted after the sample. The intensity of SHG light is quadratically proportional to the sample thickness L and appropriate $\chi^{(2)}$ susceptibility tensor element by equation[8]:

$$I_{2\omega} \propto= \left|\frac{\chi^{(2)}}{\Delta k}\right|^2 4sin^2\left(\frac{\Delta kL}{2}\right) \quad \text{(Eq. 8)}$$

where $\Delta k = k_{2\omega} - 2k_\omega$ is the **k**-vector mismatch due to $n_o(\lambda)$ refractive index dispersion. Note that when $\Delta k \neq 0$, the SHG signal is limited by the coherence length l_c and does not follow the square proportionality with a crystal thickness L.

From equation (7), it follows that the generated SHG signal intensity $I_{2\omega}$ is proportional to the effective second-order nonlinear susceptibility tensor coefficients as:

$$I_{2\omega} \propto (\chi_{111}cos\theta^2 - \chi_{111}sin\theta^2)^2 - (2\chi_{111}cos\theta sin\theta)^2. \quad \text{(Eq. 9)}$$

The measurements confirm theoretical predictions for this particular experiment when SHG intensity was measured without any linear polarizer, as demonstrated in Figure S8.

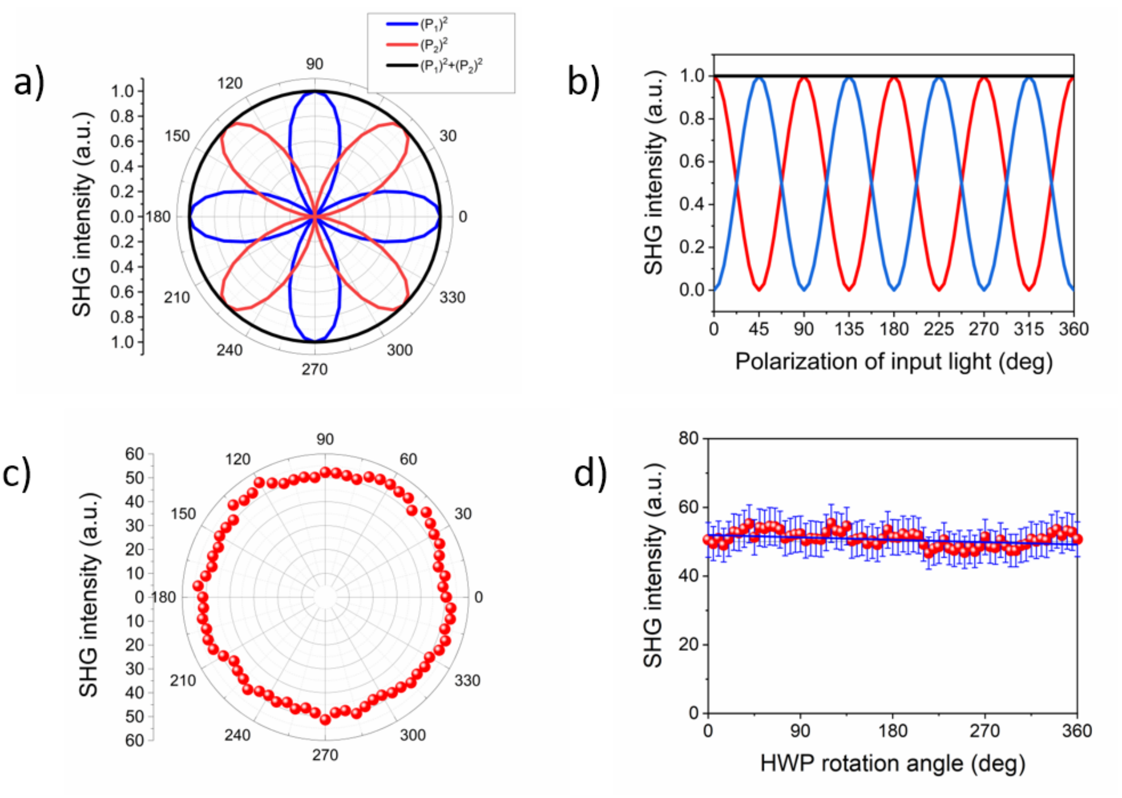

Figure S7. Theoretical predictions of expected shapes of SHG intensity for **TMB** crystal upon rotation of linear polarization of Nd:YAG excitation beam: a) polar plot, circular shape (black line) of SHG intensity is expected when no polarizer for generated SHG light is used, blue line corresponds to the measurement of $(P_1)^2$ and red line to the $(P_2)^2$ component of SHG intensity. b) The same result as in (a) plotted in a linear plot with respect to HWP rotation angle. c) Experimental result of SHG intensity obtained with full rotation of half-wave plate, i.e., incident linear polarization rotation by 360°, on polar plot the circular shape has been measured. d) SHG intensity versus HWP rotation angle in a linear plot with respect to HWP rotation angle.

The equations (7) and (9) correctly describe the SHG process in dipolar approximation when linear incident light polarization is rotated by 360°.

1.5 Nonlinear chiroptic effect: SHG -CD modelling

Numerical analysis of SHG response of **TMB** single crystal under irradiation with linearly polarized excitation laser beam from both sides of crystal plate and assuming QWP constant rotation requires assumption of some simplifications. We treated the unknown chiroptical SHG response by adding a chiral term in the form: $\pm[\Delta\chi^{chiral}+\chi^{eem}\sin(2\phi)]$ with angle ϕ corresponding to the rotation of QWP, $\Delta\chi^{chiral}$ corresponds to chiral difference susceptibility, χ^{eem} describes all effects of SHG generation due to dipole-magnetic coupling, and ± signs correspond to two possible **c**-axis orientations with respect to the incident excitation beam direction. First term $\Delta\chi^{chiral}$ introduces the intrinsic crystal chirality observed as SHG-CD. Next this chiral term was added to the second order polarizations $P_x(2\omega)$ and $P_y(2\omega)$ with a 0.5 weight. Then, to calculate angular dependence of SHG on QWP rotation angle ϕ the squares of these functions were plotted in a linear and polar plots with assumption that $d_{11} = 1$ and electric field amplitude E =

1 what allows to generate the normalized SHG-CD response, free of magnitude of nonlinear coefficient and electric field amplitude of excitation laser light. The summary results of this approach are shown in Figs. S9 and S10. Comparison of results of calculations of chiral contribution for the **TMB** crystal with positive and negative chiral axis direction with respect to the incoming laser beam shows that indeed two different shapes of polarimetric plots can be observed. The estimation of SHG-CD according to a definition proposed by eq. (2) in main manuscript in this case is not obvious because minima and maxima of SHG signals are shifted from + 45° and + 135° for which the SHG-CD is measured. These shifts depend on the values of nonlinear susceptibilities χ^{eee}, crystal symmetry and excitation beam incidence angle with respect to the chiral ***c***-axis. The chosen values for the chiral contribution function allowed us to find out the similar polarimetric shapes to those measured experimentally, of course disregarding the absolute values of SHG intensities obtained in the experiments.

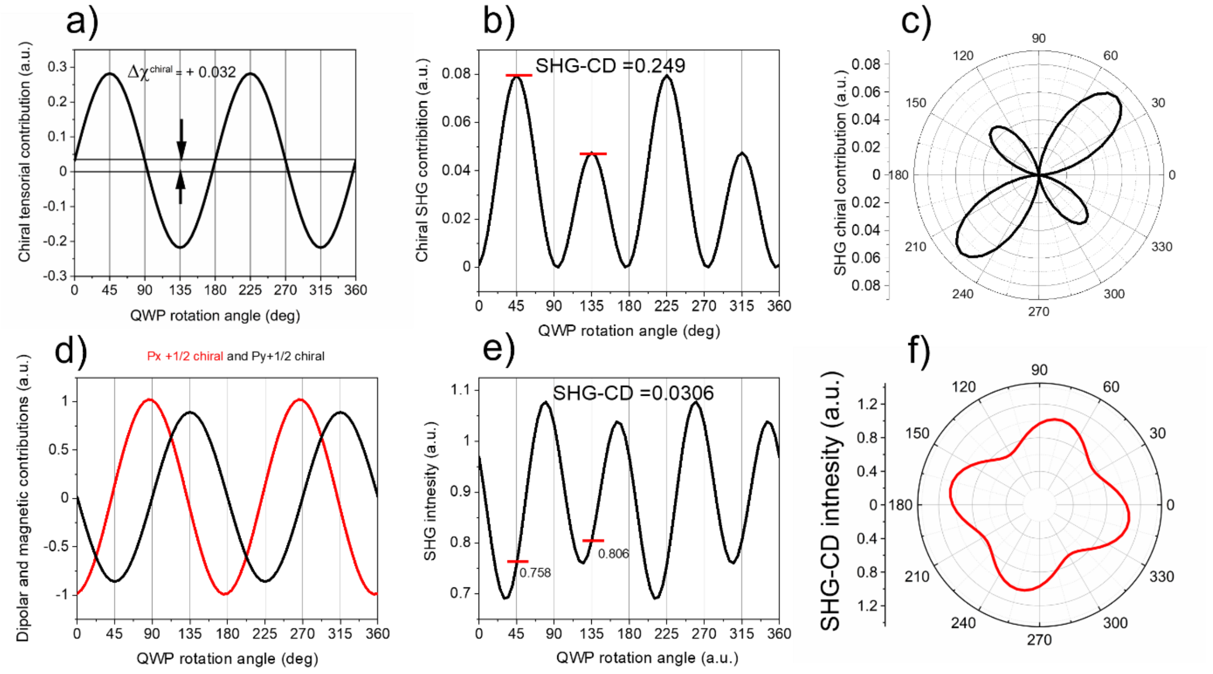

Figure S8. Methodology of creating the expected shape of SHG-CD in **TMB** single crystal with positive $\Delta\chi^{chiral}$ = 0.032 and χ^{eem} = 0.25. a) function [0.032+0.25 sin(2ϕ)] versus QWP rotation angle ϕ; b) function $[0.032+0.25\ \sin(2\phi)]^2$, with SHG-CD = 0.249; c) polarimetric plot of chiroptic contribution to SHG; d) plots of $P_x(2\omega) + 0.5[0.032+0.25\ \sin(2\phi)]$ and $P_y(2\omega) + 0.5[0.032+0.25\ \sin(2\phi)]$; e) plot of $[P_x(2\omega) + 0.5[0.032+0.25\ \sin(2\phi)]]^2 + [P_y(2\omega) + 0.5[0.032+0.25\ \sin(2\phi)]]^2$, here SHG-CD = 0.0306 and f) polarimetric plot of the last function.

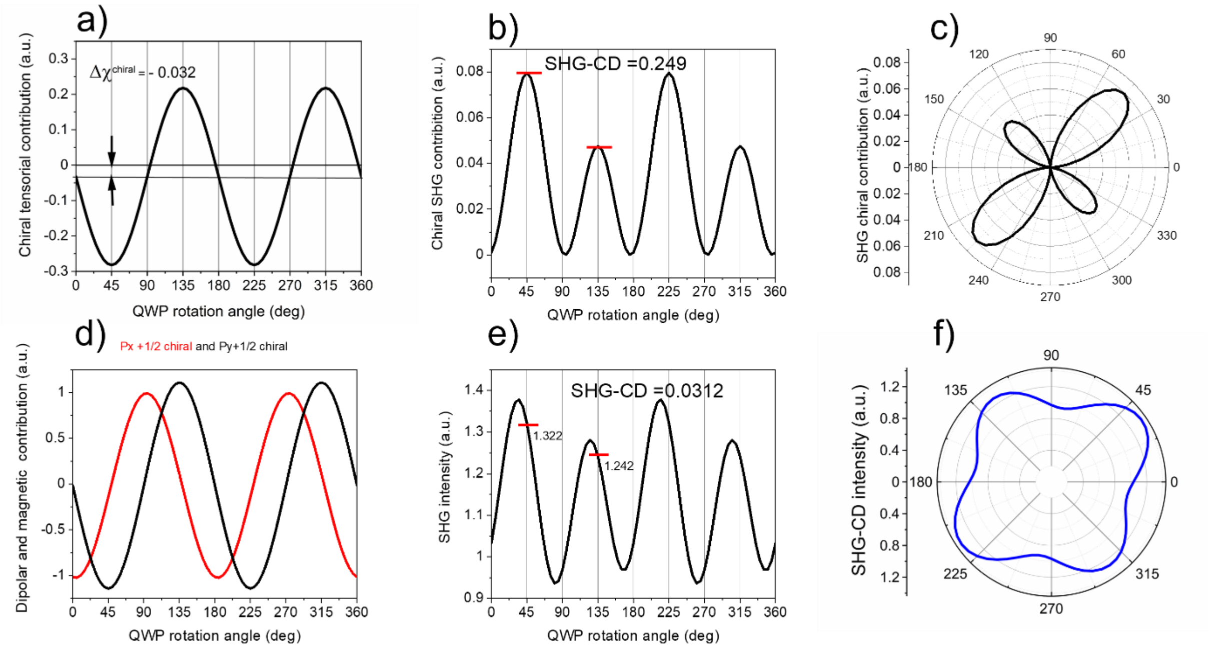

Figure S9. Methodology of creating the expected shape of SHG-CD in **TMB** single crystal with negative $\Delta\chi^{chiral}$ = – 0.032 and χ^{eem} = – 0.25. a) function – [0.032+0.25 sin(2ϕ)] versus QWP rotation angle ϕ; b) function – $[0.032+0.25 \sin(2\phi)]^2$, with SHG-CD = 0.249; c) polarimetric plot of chiroptic contribution to SHG; d) plots of $P_x(2\omega) - 0.5[0.032+0.25 \sin(2\phi)]$ and $P_y(2\omega) - 0.5[0.032+0.25 \sin(2\phi)]$; e) plot of $[P_x(2\omega) - 0.5[0.032+0.25 \sin(2\phi)]]^2 + [P_y(2\omega) - 0.5[0.032+0.25 \sin(2\phi)]]^2$, here SHG-CD = 0.0312 and f) polarimetric plot of the last function.

To correctly reproduce the measured shapes of SHG-CD we assumed the net chiral contribution to SHG at the level of 25% of the nonchiral one. However, the SHG-chiral difference as given by the equation (2) of the main manuscript is much smaller and in the considered case amounts merely to $\Delta\chi^{chiral} \sim 3.1\%$.

1.6 Photoluminescence properties

The absorption spectra were measured at room temperature on a Varian Cary 5000 Scan spectrophotometer in the range of 300–900 nm. The measurements of emission and excitation spectra, photo-luminescence quantum yields (PLQY) and decay times were performed at room temperature on an Edinburgh Instruments FLS980 spectrofluorometer equipped both with a xenon and microsecond lamp. During measurements, optical filters were used. Using an integrating sphere from Edinburgh Instruments with a small elliptical mirror and a baffle plate for beam direction and protection against directly incident light, quantum yield measurements were carried out. The standard sample holder inside the sample chamber was swapped out for the integrating sphere during the measurement. The software provided by Edinburgh Instruments was used to calculate photoluminescence quantum yields.

The plots showing the luminescence decay curves for **TMB** powder and crystals are shown in Figure S10.

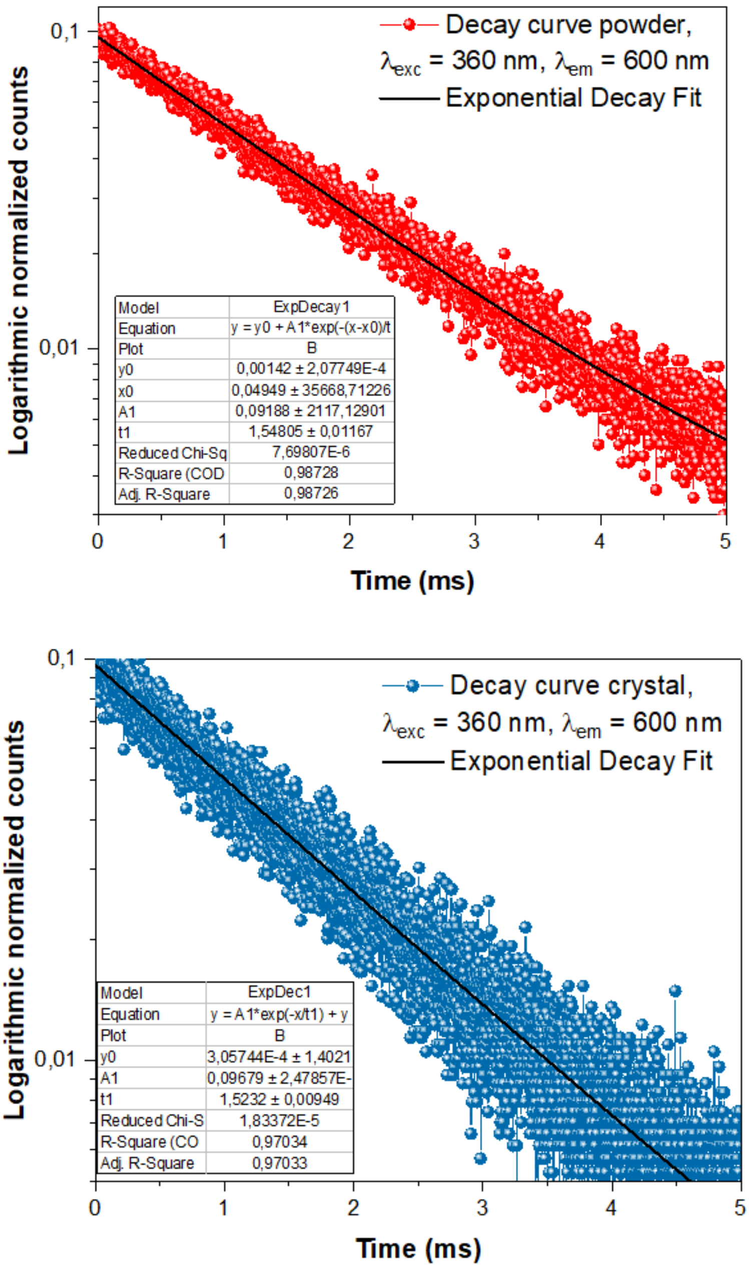

Figure S10. Luminescence decay times measured for a) powder sample; b) crystal sample of **TMB**.

2. References

(1) *CrysAlis RED, CrysAlis CCD,Oxford Diffraction:*; Oxford Diffraction Ltd, Abingdon, England, **2008**.

(2) Sheldrick, G. M. Crystal Structure Refinement with SHELXL. *Acta Crystallogr. Sect. C Struct. Chem.* **2015**, *71* (Md), 3–8.

(3) MacRae, C. F.; Sovago, I.; Cottrell, S. J.; Galek, P. T. A.; McCabe, P.; Pidcock, E.; Platings, M.; Shields, G. P.; Stevens, J. S.; Towler, M.; et al. Mercury 4.0: From Visualization to Analysis, Design and Prediction. *J. Appl. Crystallogr.* **2020**, *53*, 226–235.

(4) De Jong, M.; Chen, W.; Geerlings, H.; Asta, M.; Persson, K. A. A Database to Enable Discovery and Design of Piezoelectric Materials. *Sci. Data* **2015**, *2*, 1–13.

(5) Kurtz, S. K.; Perry, T. T. A Powder Technique for the Evaluation of Nonlinear Optical Materials. *J. Appl. Phys.* **1968**, *39* (8), 3798–3813.

(6) Dougherty, J. P.; Kurtz, S. K. A Second Harmonic Analyzer for the Detection of Non-Centrosymmetry. *J. Appl. Cryst* **1976**, *9*, 14–158.

(7) Eckardt, R. C.; Masuda, H.; Fan, Y. X.; Byer, R. L. Absolute and Relative Nonlinear Optical Coefficients of KDP, KD∗P, BaB2O4, LiIO3, MgO:LiNbO3, and KTP Measured by Phase Matched Second-Harmonic Generation. *IEEE J. Quantum Electron.* **1990**, *26*, 922–933.

(8) Boyd, R. W. Nonlinear Optics, Third Edition. In *The Institute of Optics University of Rochester Rochester, New York USA*; Academic Press is an imprint of Elsevier, **2009**; Vol. 14, p 029902.
