# Supplementary material for: Nonlinear Optical Activity of a Chiral Organic–Inorganic ([(NH3CH2CH2)3NH])2[MnBr5]Br5 Photoluminescent and Piezoelectric Crystal
Source: J Phys Chem Lett. 2024 May 9;15(19):5276–87. doi: 10.1021/acs.jpclett.4c00709 (PMC11103696; doi:10.1021/acs.jpclett.4c00709)

checkCIF/PLATON report

Structure factors have been supplied for datablock(s) 100K

THIS REPORT IS FOR GUIDANCE ONLY. IF USED AS PART OF A REVIEW PROCEDURE FOR PUBLICATION, IT SHOULD NOT REPLACE THE EXPERTISE OF AN EXPERIENCED CRYSTALLOGRAPHIC REFEREE.

No syntax errors found.   CIF dictionary   Interpreting this report

Datablock: 100K

Bond precision: C-C = 0.0060 A   Wavelength=0.71073

Cell:	a=9.8125(2)	b=9.8125(2)	c=28.643(1)
	alpha=90	beta=90	gamma=120
Temperature:	100 K		

	Calculated	Reported
Volume	2388.41(13)	2388.41(13)
Space group	R 3 2	R 3 2 :H
Hall group	R 3 2"	R 3 2"
Moiety formula	2(C6 H22 N4), Br5 Mn, 5(Br)	2(C6H22N4),Br5 Mn,5(Br)
Sum formula	C12 H44 Br10 Mn N8	C12 H44 Br10 Mn N8
Mr	1154.49	1154.59
Dx,g cm-3	2.408	2.408
Z	3	3
Mu (mm-1)	12.980	12.980
F000	1641.0	1641.0
F000’	1634.95	
h,k,lmax	12,12,35	12,12,35
Nref	1054[625]	988
Tmin,Tmax	0.514,0.523	0.772,1.000
Tmin’	0.504	

Correction method= # Reported T Limits: Tmin=0.772 Tmax=1.000
AbsCorr = MULTI-SCAN

Data completeness= 1.58/0.94   Theta(max)= 25.963

R(reflections)= 0.0143(944)   wR2(reflections)= 0.0343(988)

S = 1.051   Npar= 50

The following ALERTS were generated. Each ALERT has the format
test-name_ALERT_alert-type_alert-level.
Click on the hyperlinks for more details of the test.

Alert level C

```
PLAT042_ALERT_1_C Calc. and Reported MoietyFormula Strings  Differ     Please Check
PLAT094_ALERT_2_C Ratio of Maximum / Minimum Residual Density ....       2.10 Report
PLAT711_ALERT_1_C BOND    Unknown or Inconsistent Label .........          4 Check
                    C3          4
```

Alert level G

```
PLAT007_ALERT_5_G Number of Unrefined Donor-H Atoms .............          3 Report
PLAT017_ALERT_1_G Check Scattering Type Consistency of 3A       as         H
PLAT304_ALERT_4_G Non-Integer Number of Atoms in ..... (Resd  3  )       0.50 Check
PLAT304_ALERT_4_G Non-Integer Number of Atoms in ..... (Resd  4  )       0.33 Check
PLAT480_ALERT_4_G Long H...A H-Bond Reported H2A      ..BR3      .       3.01 Ang.
PLAT480_ALERT_4_G Long H...A H-Bond Reported H2B      ..BR3      .       3.07 Ang.
PLAT480_ALERT_4_G Long H...A H-Bond Reported H3B      ..BR1      .       2.97 Ang.
PLAT720_ALERT_4_G Number of Unusual/Non-Standard Labels .........          1 Note
PLAT764_ALERT_4_G Overcomplete CIF Bond List Detected (Rep/Expd) .       1.29 Ratio
PLAT794_ALERT_5_G Tentative Bond Valency for Mn1       (II)      .       2.46 Info
PLAT883_ALERT_1_G No Info/Value for _atom_sites_solution_primary .     Please Do !
PLAT899_ALERT_4_G SHELXL97  is Deprecated and Succeeded by SHELXL-     2019/2 Note
PLAT961_ALERT_5_G Dataset Contains no Negative Intensities .......     Please Check
PLAT965_ALERT_2_G The SHELXL WEIGHT Optimisation has not Converged     Please Check
PLAT967_ALERT_5_G Note: Two-Theta Cutoff Value in Embedded .res ..       52.0 Degree
PLAT978_ALERT_2_G Number C-C Bonds with Positive Residual Density.          1 Info
```

0 **ALERT level A** = Most likely a serious problem - resolve or explain
0 **ALERT level B** = A potentially serious problem, consider carefully
3 **ALERT level C** = Check. Ensure it is not caused by an omission or oversight
16 **ALERT level G** = General information/check it is not something unexpected

4 ALERT type 1 CIF construction/syntax error, inconsistent or missing data
3 ALERT type 2 Indicator that the structure model may be wrong or deficient
0 ALERT type 3 Indicator that the structure quality may be low
8 ALERT type 4 Improvement, methodology, query or suggestion
4 ALERT type 5 Informative message, check

It is advisable to attempt to resolve as many as possible of the alerts in all categories. Often the minor alerts point to easily fixed oversights, errors and omissions in your CIF or refinement strategy, so attention to these fine details can be worthwhile. In order to resolve some of the more serious problems it may be necessary to carry out additional measurements or structure refinements. However, the purpose of your study may justify the reported deviations and the more serious of these should normally be commented upon in the discussion or experimental section of a paper or in the "special_details" fields of the CIF. checkCIF was carefully designed to identify outliers and unusual parameters, but every test has its limitations and alerts that are not important in a particular case may appear. Conversely, the absence of alerts does not guarantee there are no aspects of the results needing attention. It is up to the individual to critically assess their own results and, if necessary, seek expert advice.

Publication of your CIF in IUCr journals

A basic structural check has been run on your CIF. These basic checks will be run on all CIFs submitted for publication in IUCr journals (*Acta Crystallographica*, *Journal of Applied Crystallography*, *Journal of Synchrotron Radiation*); however, if you intend to submit to *Acta Crystallographica Section C* or *E* or *IUCrData*, you should make sure that full publication checks are run on the final version of your CIF prior to submission.

Publication of your CIF in other journals

Please refer to the *Notes for Authors* of the relevant journal for any special instructions relating to CIF submission.

PLATON version of 28/11/2022; check.def file version of 28/11/2022

Datablock 100K - ellipsoid plot

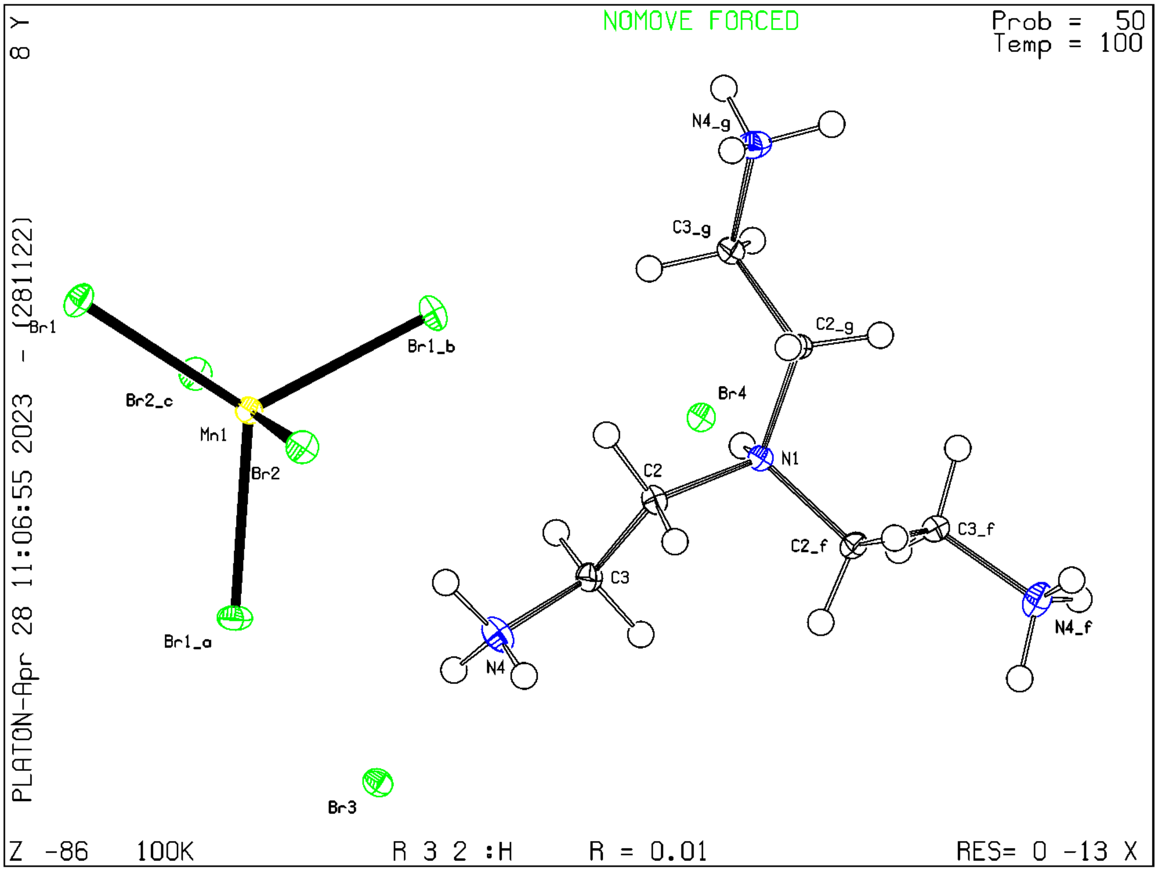

Supplement: Supplementary file 3 — jz4c00709_si_003.pdf [file jz4c00709_si_003.pdf]
