# Supplementary material for: Nonlinear Optical Activity of a Chiral Organic–Inorganic ([(NH3CH2CH2)3NH])2[MnBr5]Br5 Photoluminescent and Piezoelectric Crystal
Source: J Phys Chem Lett. 2024 May 9;15(19):5276–87. doi: 10.1021/acs.jpclett.4c00709 (PMC11103696; doi:10.1021/acs.jpclett.4c00709)
Supplement: Supplementary file 4 — jz4c00709_si_004.pdf [file jz4c00709_si_004.pdf]

jz-2024-00709w.R1

Name: Peer Review Information for "Nonlinear Optical Activity of Chiral Organicinorganic ([(NH₃CH₂CH₂)₃NH])₂[MnBr₅]Br₅ Photoluminescent and Piezoelectric Crystal"

First Round of Reviewer Comments

Reviewer: 1

Comments to the Author

Rok et al. synthesized a bulk Mn-based perovskite single crystal (TMB) with a size about 2 cm. According to SC-XRD measurement, the Mn-based perovskite crystalizes in the high symmetry, chiral space group of R32 and the nonlinear optical activity, photoluminescent and piezoelectric property were studied. The piezoelectric properties of the TMB crystal were demonstrated by the characteristic resonance curves. The second harmonic generation and the second harmonic generation circular dichroism were observed on TMB. The temperature dependent photoluminescence of TMB single crystal was also measured and a high photoluminescence quantum yields of 70% was obtained. I would like to recommend the publication of their manuscript in JPCL after the following issues being properly addressed in a major revision.

(1) The TMB crystalized in a chiral space group of R32 using a nonchiral cation, according to the literature, enantiomers of single crystal should be found by repeating the single-crystal test. It is necessary to show the structure of all enantiomers.

(2) The TMB exhibit a CD signal as shown in fig. 6b, is it a real signal? It is better to take measurements on multiple samples to ensure the authenticity of the data and verify the existence of a symmetric CD spectrum.

(3) The SHG-CD and photoluminescence would like to deeply discuss according to the previous literature (DOI: 10.1002/anie.202309600; 10.1007/s11426-024-1946-7).

(4) The author claims that the TMB have piezoelectric properties, thus the piezoelectric coefficient of TMB should be given.

(5) According to the previous work, the SHG-CD signal will be influenced by the anisotropic of crystal, it is better to measure the SHG-CD spectra with different single crystal orientation or rotating the single crystal.

(6) The most important progress in chiral perovskite is missed (DOI: 10.1038/s41566-018-0220-6; 10.1038/s41578-020-0181-5).

Reviewer: 2

Comments to the Author

In this work, Rok et al. reported the circular dichroism, second harmonic generation and photoluminescence in a Mn(II)-based organic-inorganic hybrid ([(NH3CH2CH2)3NH])2[MnBr5]Br5 (TMB). This compound has a unusual trigonal bipyramidal geometry of Mn(II) cation central polyhedra and exhibit a pretty high photoluminescence quantum yield (PLQY). However, this manuscript is badly written and lack of new physical insights, based on the following reasons:

1. As the authors mentioned, TMB has trigonal bipyramidal [MnBr5] groups. However, there was lack of any discussion on how this type of unique groups influence the nonlinear optical effect in TMB. In fact, the second harmonic generation effect in this compound is rather small.

2. The same problem occurred in the section of luminescence. The authors merely stated that the luminescent properties of trigonal bipyramidal coordination would be different with those of tetrahedral and octahedral geometries, but no detailed analysis on how the crystal field splitting due to chemical coordination affects the luminescent properties, e.g., excitation spectrum and PLQY, etc.

3. The authors did not make detailed discussion on the correlation between chiral structure and circular dichroism in TMB.

4. The whole manuscript is tedious. Many texts are common-sense introductions. The authors are needed to significantly trim up every chapter, and put the less important figures and texts in the Supplementary Materials.

In summary, this work does not provide new physical insights and cannot be accepted to publication at the current stage and major revision are required by considering the above comments and suggestions.

Reviewer: 3

Comments to the Author

The authors report on the synthesis of (C6N4H22)2[MnBr5]Br5 (TMB) crystal, which belongs to the trigonal noncentrosymmetric space group R32, and they have further studied this chiral TMB structure in terms of optical activity, piezoelectricity, and second-order generation. The results are interesting and overall convincing. I suggest the authors to consider the following issues in a revised version before the acceptance of their manuscript in JPCL.

1. The chiral structures are synthesized from nonchiral raw materials. In this case, can the chirality of TMB crystals be controlled in the preparation? In addition, Figure 4 shows the “corkscrew” motif in one unit cell, so is the “corkscrew” motif in the neighboring unit cell screwed in the same way? Otherwise, the structure will be achiral.

2. What is the structure-property relationship for piezoelectricity? I assume the audience is probably not familiar with it, so it would be better to add the discussion.

3. Why the SHG intensity in Figure 9b is twice of that in Figure 9d? I assume the intensity should not change obviously by reversing the crystal to the light beam.

4. "The measured photoluminescence quantum yield of TMB compound both in its powder and single crystal forms at room temperature is around 70%. Note that PLQY is significantly larger than that reported previously.20" A PLQY of 70% is remarkably high for these hybrid perovskites, and the authors should explain why it is significantly improved compared with the previous report.

Author's Response to Peer Review Comments:

Monday, 29 April 2024
Manuscript ID: jz-2024-00709w

Corresponding Author: dr hab. Magdalena Rok

Dear Editor,

We are grateful to all the Reviewers for their effort in carefully and critically reading the manuscript submitted to JPCL and associated ESI†. The comments and the remarks certainly helped us to make our contribution more clear and more scientifically sound.

The manuscript has been revised carefully according to the Reviewers' comments and improved by removal of some sentences and by shifting some parts of the text to the ESI†. We added 18 new references and changed the graphical abstract. The point-by-point replies to the comments are listed below. In addition, we have prepared the manuscript in two versions, one of which has the changes highlighted in red (Manuscript only for Reviewers).

According to the comments of the Referees, the following major changes were made in the paper:

Reviewer #1:

Comments:
Rok et al. synthesized a bulk Mn-based perovskite single crystal (TMB) with a size about 2 cm. According to SC-XRD measurement, the Mn-based perovskite crystalizes in the high symmetry, chiral space group of R32 and the nonlinear optical activity, photoluminescent and piezoelectric property were studied. The piezoelectric properties of the TMB crystal were demonstrated by the characteristic resonance curves. The second harmonic generation and the second harmonic generation circular dichroism were observed on TMB. The temperature dependent photoluminescence of TMB single crystal was also measured and a high photoluminescence quantum yields of 70% was obtained. I would like to recommend the publication of their manuscript in JPCL after the following issues being properly addressed in a major revision.

(1) The TMB crystalized in a chiral space group of R32 using a nonchiral cation, according to the literature, enantiomers of single crystal should be found by repeating the single-crystal test. It is necessary to show the structure of all enantiomers.

Author note:

The crystal has been measured by single-crystal X-Ray diffraction. The method cannot distinguish which enantiomer is analyzed. The structure of the second enantiomer is accessible by inverting the final model of the structure. The method allows the orientation of the crystal to be projected instantly; please see the attached figure (Fig. A). Also, please find attached cif

file of the inverted structure (the structure of the second enantiomer), with the Flack parameter of 0.996(14), suggesting the wrong absolute structure.

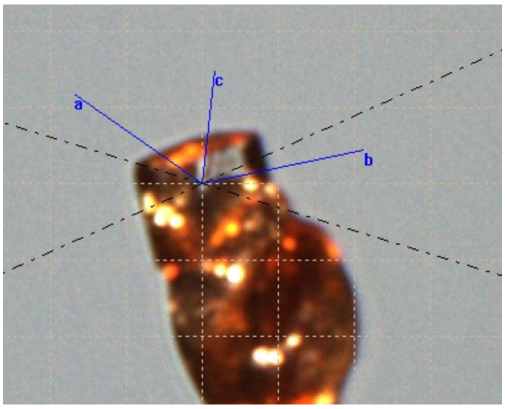

Fig. A. Orientation of crystallographic directions of the single crystal.

(2) The TMB exhibit a CD signal as shown in fig. 6b, is it a real signal? It is better to take measurements on multiple samples to ensure the authenticity of the data and verify the existence of a symmetric CD spectrum.

Author note:

This is an important question. The signal observed in Fig. 6b, according to the discussion in the main manuscript does not origin from the chirality of any single component of the crystal, both organic cation as well as bipyramids are not chiral. CD signal is the result of structural chirality of the crystal as a whole as a result of chiral 3-fold axis around which the organic cations are distributed via symmetry. Similar situation occurs in chiral nematics (cf. *J. Mater. Chem.*, **2012**, *22*, 7088-7097, DOI https://doi.org/10.1039/C2JM15962G), in which achiral molecules minimizing their interaction energy form helicoidal arrangements which are able to rotate linear polarization of light, i.e. show optical activity. CD signal which we presented in this work in Fig. 6 is known in literature as “apparent circular dichroism”. We discuss its origin and cite relevant publications. At present we have no available good quality samples to perform new CD measurements, but we check that the signal is repeatable, changes its sign under crystal inversion with respect to incoming light and on tilting the crystal by +/ - 30 deg, the signal drops significantly, what we show in Fig. S3 of ESI†. Please also read the information about this in chapter 1.4.1 Circular dichroism in TMB crystal of ESI†.

In the main manuscript on page 11 we put the sentence: “It must be noted that an “apparent CD” is dependent on a sample thickness and the crystal orientation with respect to the light incidence direction. Tilting off the crystal by 30° in a CD spectrometer caused significant drop of the “apparent CD” (see Fig. S3 of ESI†).

(3) The SHG-CD and photoluminescence would like to deeply discuss according to the previous literature (DOI: 10.1002/anie.202309600; 10.1007/s11426-024-1946-7).

Author note:

Thank you for mentioning the two important papers. We include them into our reference list. However, the described in these publications' cases are much different from ours. In *Angew. Chem. Int. Ed.* **2023**, *62* the space group is $P2_1$ and chiral cations are used to promote chirality, while in Science China-Chemistry, **2024** the space group is $P1$ and also the chirality is transferred from chiral cations. Moreover, none of the compounds contains Mn^{2+} ion. Our discussion is different because the chirality is not induced by chiral molecule and the space group is also different R32. This makes that the CD spectra are much different and discussion cannot be based on chiral cations. In our work we have proposed the modelling of SHG-CD in different way due to different crystal symmetry, but generally the results are quite similar. In our work we point out that chiroptical effects are dependent on geometry of experiment and universal measures of chirality are sometime hard to asses and hard to define by simple anisotropy factor $g_{SHG\text{-}CD.}$ Because we did not perform quantum chemical calculations of the bands structure of the studied compound and any calculations of second order susceptibilities we are unable to deepened the discussion. But we hope that this can be done in the future.

(4) The author claims that the TMB have piezoelectric properties, thus the piezoelectric coefficient of TMB should be given.

Author note:

We devoted the chapter 1.3.2 "Piezoelectric effect" in ESI†, to comment about the piezoelectricity in the studied crystal. The measurement of the piezoelectric effect is not a simple task as it requires large crystal and a few proper precision cuts, so it is a subject for a separate publication. The only relatively easy applied method in our case of platelet shape of TMB crystal would be the method based on AFM, when a conducting tip being in contact with the crystal surface is making stress generating surface charge. The drawback of this method (available in our laboratory) is that it is limited to accurately measure only the d_{333} piezoelectric coefficient, which due to symmetry reasons is absent in the studied crystal. The remark about piezoelectricity has been addressed also by Reviewer #3, remark 2, so please refer to our response to this Reviewer. We are citing here also one sentence: "From the work of de Jong, et al. and Fig. 6 therein [de Jong, M.; Chen, W.; Geerlings, H.; Asta, M.; Persson, K. A. A database to enable discovery and design of piezoelectric materials. *Sci Data* **2015**, *2*, 150053.] it follows that in the chiral point group 32 the piezoelectric tensor $\|e_{ijk}^{T}\|$ largest values do not exceed 2.5 C/m^2 for any until now measured crystal." (ESI, p.S7)

In the main manuscript on page 10 we add the following sentence:

"We simply observe that at certain crystal temperatures the used frequency of *AC* electric field matches perfectly with the mechanical crystal resonance (local $\varepsilon'(T)_{max}$) followed by the

associated anti-resonance (local $\varepsilon'(T)_{min}$) as is clearly seen in Fig. 5a. The additional information about the piezoelectric effect in chiral space group *R*32 is described in ESI† chapter 1.3.2."

(5) According to the previous work, the SHG-CD signal will be influenced by the anisotropic of crystal, it is better to measure the SHG-CD spectra with different single crystal orientation or rotating the single crystal.

Author note:

Thank you for this comment. Obviously, it is correct that the SHG-CD signal will be influenced by the anisotropy of the crystal. The natural large face of the **TMB** crystal is perpendicular to the chiral c-axis, therefore for the simplicity of calculations we chose this preferential geometry. Any departure of the **k**-vector of incident light with respect to the ***c***-axis produced asymmetric polarimetric response in SHG-CD signal. Therefore, we put a lot of effort to properly align the crystal with respect to incoming 1064 nm laser light beam and we resign from focusing incoming light to avoid spurious SHG signal connected to crystal birefringence. Please note that the crystal is optically uniaxial with optical axis (extraordinary refractive index) along the ***c***-axis, by symmetry reasons. Similar problems we faced when linear effect of CD dichroism was measured. In this case, we performed several experiments in turning the crystal in the holder with respect to the incoming light and the CD signal was sensitive to the tilt angle as expected. SHG-CD will be absent at any direction of laser IR light beam being perpendicular to the crystal ***c***-axis.

(6) The most important progress in chiral perovskite is missed (DOI: 10.1038/s41566-018-0220-6; 10.1038/s41578-020-0181-5).

Author note:

Thank you for this important remark. Yes, in fact we missed in our discussion these very important publications, which enlarged our knowledge about possible applications of chiral crystals.

In the revised version of our manuscript we put a following text on page 5:

"The chiroptical and chiral-related electric and magnetic properties have recently attracted very wide attention due to both scientific interest as well as potential applications in various fields (see the recently published review publications and references therein)[32–36]. Long et al.[32] in the excellent review concerning research and applications of chiral-perovskites in optoelectronics gives the broad outlook on different aspects of these very promising materials. Among the unique achievements one can mention reports on spin control in reduced-dimensional chiral perovskites[37], their topological quantum properties[38], spintronics, report on chiral- perovskite photodetectors with responsivity 100 times higher than that of chiral metasurface photodetectors[39,40] and third order nonlinear optical effect of two-photon absorption

upconverted circularly polarized fluorescence in chiral perovskite nanocrystals[40], just to mention few examples”

(32) Long, G.; Sabatini, R.; Saidaminov, M. I.; Lakhwani, G.; Rasmita, A.; Liu, X.; Sargent, E. H.; Gao, W. Chiral-Perovskite Optoelectronics. *Nat. Rev. Mater.* **2020**, *5*, 423–439.
(33) Ma, J.; Wang, H.; Li, D. Recent Progress of Chiral Perovskites: Materials, Synthesis, and Properties. *Adv. Mater.* **2021**, *33*, 20200875 (1-22).
(34) Ma, S.; Ahn, J.; Moon, J. Chiral Perovskites for Next-Generation Photonics: From Chirality Transfer to Chiroptical Activity. *Adv. Mater.* **2021**, *33*, 2005760 (1-19).
(35) Pietropaolo, A.; Mattoni, A.; Pica, G.; Fortino, M.; Schifino, G.; Grancini, G. Rationalizing the Design and Implementation of Chiral Hybrid Perovskites. *Chem* **2022**, *8*, 1231–1253.
(36) Dang, Y.; Liu, X.; Cao, B.; Tao, X. Chiral Halide Perovskite Crystals for Optoelectronic Applications. *Matter* **2021**, *4*, 794–820.
(37) Long, G.; Jiang, C.; Sabatini, R.; Yang, Z.; Wei, M.; Quan, L. N.; Liang, Q.; Rasmita, A.; Askerka, M.; Walters, G.; et al. Spin Control in Reduced-Dimensional Chiral Perovskites. *Nat. Photonics* **2018**, *12*, 528–533.
(38) Sanchez, D. S.; Belopolski, I.; Cochran, T. A.; Xu, X.; Yin, J. X.; Chang, G.; Xie, W.; Manna, K.; Süß, V.; Huang, C. Y.; et al. Topological Chiral Crystals with Helicoid-Arc Quantum States. *Nature* **2019**, *567*, 500–505.
(39) Wang, J.; Zhang, C.; Liu, H.; McLaughlin, R.; Zhai, Y.; Vardeny, S. R.; Liu, X.; McGill, S.; Semenov, D.; Guo, H.; et al. Spin-Optoelectronic Devices Based on Hybrid Organic-Inorganic Trihalide Perovskites. *Nat. Commun.* **2019**, *10*, 1–6.
(40) Chen, C.; Gao, L.; Gao, W.; Ge, C.; Du, X.; Li, Z.; Yang, Y.; Niu, G.; Tang, J. Circularly Polarized Light Detection Using Chiral Hybrid Perovskite. *Nat. Commun.* **2019**, *10*, 1–7.

Reviewer #2:

In this work, Rok et al. reported the circular dichroism, second harmonic generation and photoluminescence in a Mn(II)-based organic-inorganic hybrid ([(NH3CH2CH2)3NH])2[MnBr5]Br5 (TMB). This compound has an unusual trigonal bipyramidal geometry of Mn(II) cation central polyhedra and exhibit a pretty high photoluminescence quantum yield (PLQY). However, this manuscript is badly written and lack of new physical insights, based on the following reasons:

1. As the authors mentioned, TMB has trigonal bipyramidal [MnBr5] groups. However, there was lack of any discussion on how this type of unique groups influence the nonlinear optical effect in TMB. In fact, the second harmonic generation effect in this compound is rather small.

Author note:

According to theory, the SHG efficiency depends on highly polarizable parts of molecules or atoms assembles. The nonlinear response is usually calculated considering contribution of all higher energy levels, the ones which counts more are that which are closer to sum of energy of two incoming photons, i.e. $2\hbar\omega$, due to so called near resonance effect. In the described case energy levels of cations are much higher than energy terms of Mn^{+2}. The exact response could only be obtained by quantum chemical calculations, which is a difficult task due to hybrid nature of the studied compound.

We add on page 16 of the main manuscript the discussion regarding the strength of nonlinear optical effect (SHG) in relation to the structure of the studied compound:

“The relatively low value of nonlinear optical tensor coefficients in **TMB** can be rationalized by the crystallographic structure analysis. Organic molecules have demonstrated their unique properties for the nonlinear optics due to large values of their first hyperpolarizabilities β_{ijk},

mostly due to delocalized cloud of π electrons (e.g. "push-pull" or D-π–A architecture systems). In the studied compound the organic H_4*tren* cation does not contain any extended π electronic system. It rather represents the nondipolar molecule known as octupolar one. All dipolar-like quantities, i.e. the dipole moment μ_0, and the vector part of β_{iii}, vanish for a purely octupolar molecule and only the symmetry-allowed octupolar components can contribute to second-order NLO effects.[67] Therefore, the second order NLO effects are not large. The contribution from the inorganic part could arise from the field induced asymmetry of trigonal bipyramidal $[MnBr_5]^{3-}$ groups. However, without detailed quantum-chemical calculations it is difficult to judge how large contribution to SHG susceptibilities $\chi^{(2)}$ they can add.[68] The role of the N-H···Br hydrogen bonds linking $[MnBr_5]^{3-}$ anions with cations could not be prevailing due to the weakness of these bonds (see Table S3 of ESI†).

(67) J. Jerphagnon, D. C.; Bonneville, R. The Description of the Physical Properties of Condensed Matter Using Irreducible Tensors. *Adv. Phys.* **1978**, *27*, 609–650.

(68) Bergman, J. G.; Crane, G. R. Structural Aspects of Nonlinear Optics: Optical Properties of KIO2F2 and Its Related Iodates. *J. Chem. Phys.* **1974**, *60*, 2470–2474.

2. The same problem occurred in the section of luminescence. The authors merely stated that the luminescent properties of trigonal bipyramidal coordination would be different with those of tetrahedral and octahedral geometries, but no detailed analysis on how the crystal field splitting due to chemical coordination affects the luminescent properties, e.g., excitation spectrum and PLQY, etc.

Author note:

As suggested by the Reviewer, the following information has been added on page 24 and 25 of the main manuscript regarding luminescent properties in **TMB** crystal.

"All of the optical absorption transitions for Mn^{2+} observed in excitation spectra are forbidden by both parity and spin rules.[75,81] A characteristic feature of the Mn^{2+} excitation spectrum is the varying spectral widths of the absorption transitions. The effect is also predicted by Tanabe-Sugano diagrams[76], as a result of the strength of the crystal field changes during atomic vibrations and of temperature changes that control the lattice phonons energy. Generally, the lowest energy absorption and excitation bands of Mn^{2+} located in the range of 400 – 520 nm are of low-intensities. This causes disadvantages in potential applications for manganese-based materials. However, great effort was put into improving the emission intensity.[82] In the case of the crystal in this study, it was possible to obtain material with high photoluminescence quantum yield. According to TS diagram[76,81], this accounts for all the observed spectral properties: the broad luminescence band arises from varying slopes of the energy levels, the prolonged decay time results from the spin selection rule, and the emission color varies with the host lattice coordination due to its dependence on the crystal field strength effect on Mn^{2+} ion. In a tetrahedral coordination, where Mn^{2+} experiences a weak crystal field, the emission is

typically green. Conversely, in an octahedral coordination, with a stronger crystal field, Mn^{2+} emits an orange to red color.[83]"

(75) Zhou, Q.; Dolgov, L.; Srivastava, A. M.; Zhou, L.; Wang, Z.; Shi, J.; Dramićanin, M. D.; Brik, M. G.; Wu, M. Mn2+ and Mn4+ Red Phosphors: Synthesis, Luminescence and Applications in WLEDs. A Review. *J. Mater. Chem. C* **2018**, *6*, 2652–2671.

(76) Tanabe, Y.; Sugano, S. On the Absorption Spectra of Complex Ions, III The Calculation of the Crystalline Field Strength. *J. Phys. Soc. Japan* **1956**, *11*, 864–877.

(81) Blasse, G.; Grabmaier, B. C. A General Introduction to Luminescent Materials. In *Luminescent Materials*; Springer Berlin Heidelberg: Berlin, Heidelberg, 1994; pp 1–9.

(82) Moon, T.; Hong, G. Y.; Lee, H. C.; Moon, E. A.; Jeoung, B. W.; Hwang, S. T.; Kim, J. S.; Ryu, B. G. Effects of Eu2+ Co-Doping on Vuv Photoluminescence Properties of BaMgAl10O17: Mn2+ Phosphors for Plasma Display Panels. *Electrochem. Solid-State Lett.* **2009**, *12*, J61–J63.

(83) Adachi, S. Review—Photoluminescence Spectroscopy of Mn2+ -Activated Phosphors: Part I. Fundamentals. *ECS J. Solid State Sci. Technol.* **2023**, *12*, 126003 (1-31)

3. The authors did not make detailed discussion on the correlation between chiral structure and circular dichroism in TMB.

<u>Author note:</u>

CD signals are inherently weak in molecules exhibiting natural optical activity. Usually, molecular CD signals are 10^{-6} to 10^{-2} of conventional absorption. This extreme weakness of chiroptical signals makes it difficult to obtain structural information with high precision. Still more difficult is the case when the chiral crystal does not contain chiral molecules or chiral atomic arrangements. The chirality in the studied TMB crystal comes from the delicate differences in the mutual positions of the three symmetry related positions of H_4*tren* cations around 3_1-axis. In the figure pasted below the structure is seen perpendicular to the ***c***-axis, the three cations are schematically visualised by ellipses, and circle with yellow arrows shows left-hand helix.

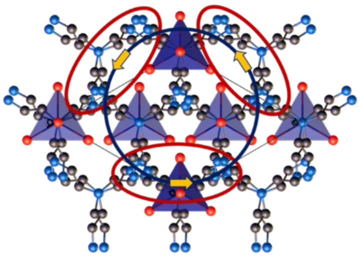

Moreover, any clear differences in absorption spectra characteristic to Mn^{2+} energy terms are not explicitly observed in CD spectra. Therefore, we think that the circular dichroism is related only to organic molecules and not to the trigonal bipyramids. Seen on the Fig. 6b inset sum of two measured CD spectra for front and rear side of the crystal should in principle remove the "apparent dichroism" at 239 nm. However, some residual signal is left which origin is not sure. We suspect, that its origin comes from the possible differences in mounting the sample in our holder and illumination of not exactly the same area in both experiments. As we mention in discussion measurements of CD in crystals is always loaded with some uncertainty.

4. The whole manuscript is tedious. Many texts are common-sense introductions. The authors are needed to significantly trim up every chapter, and put the less important figures and texts in the Supplementary Materials.

Author note:

In accordance with the Reviewer's suggestion, we remove the common-sense introductions, we remove some obvious sentences, the section on the description of the measurement of SHG and SHG-CD has been shortened, and the deleted sections have been moved to ESI†.

In summary, this work does not provide new physical insights and cannot be accepted to publication at the current stage and major revision are required by considering the above comments and suggestions.

Author note:

We understand the Reviewer's criticism that the structure and emission properties have already been published in 2017 (Polyhedron, DOI: 10.1016/j.poly.2016.10.047), what we mentioned in our paper. However, we believe the authors presented only a brief structural analysis and an emission spectrum was measured at room temperature only in the abovementioned paper. So, we treated this work as a preliminary one for further analysis. In the our manuscript proposed, we present: 1. a more detailed structural analysis, 2. dielectric measurements at different frequencies and in function of temperature to prove that the crystal has piezoelectric properties, 3. an in-depth analysis of nonlinear optical SHG and chiral SHG-CD properties (experimental results supported by theoretical tensorial calculations for the R32 space group not reported previously), 4. temperature dependence of luminescence excitation spectra, luminescence decay times for a powder and crystal, and improved photoluminescence quantum yield (PLQY) determined by an integrating sphere. All measurements were made on an oriented monocrystal, which is unique, and on a polycrystalline sample for comparison.

Reviewer #3:

The authors report on the synthesis of (C6N4H22)2[MnBr5]Br5 (TMB) crystal, which belongs to the trigonal noncentrosymmetric space group R32, and they have further studied this chiral TMB structure in terms of optical activity, piezoelectricity, and second-order generation. The results are interesting and overall convincing. I suggest the authors to consider the following issues in a revised version before the acceptance of their manuscript in JPCL.

1. The chiral structures are synthesized from nonchiral raw materials. In this case, can the chirality of TMB crystals be controlled in the preparation? In addition, Figure 4 shows the “corkscrew” motif within single unit cell, so is the “corkscrew” motif in the neighbouring unit cell screwed in the same way? Otherwise, the structure will be achiral.

Author note:

In the case of the **TMB** crystal under study, both the organic component and the inorganic part are not chiral compounds. Chirality arises spontaneously during crystallization as a result of weak hydrogen bonds that minimize the packing energy of organic ligand and inorganic bipyramids and Br atoms. We think that chirality in this case cannot be controlled. The “corkscrew” motif is the same for the whole

structure and all unit cells, as the structure of a crystal is composed of a repeated by translation vectors (***a***, ***b***, ***c***) unit cells in three dimensions.
We put the following sentence in the main manuscript on page 4 and 5: "Note that chirality of this compound is neither imparted by incorporation of any chiral organic molecules nor induced by tuning the environmental conditions, e.g. by using chiral solvents or external stimuli (such as strain)."

2. What is the structure-property relationship for piezoelectricity? I assume the audience is probably not familiar with it, so it would be better to add the discussion.

Author note:

Due to the fact that we did not study the piezoelectric effect and merely we noted its presence by observation of piezoelectric resonances during measurements of complex electric permittivity, we did not discuss structure-relationship property in this case. Instead we prepared a kind of tutorial text about piezoelectric effect in R32 chiral space group and put it to ESI† in chapter 1.3.2. :
"Piezoelectricity is the ability of electric charge accumulation in non-centrosymmetric solid materials in response to the applied mechanical stress or strain. Stress and strain are related with electric field via third order tensor:

$$e_{ijk}^{T} = \left(\frac{\partial D_i}{\partial \epsilon_{jk}}\right)_{E,T} = -\left(\frac{\partial \sigma_{jk}}{\partial E_i}\right)_{\epsilon,T}$$

where D, E, ϵ, σ and T represent the electric displacement field, the electric field, the strain tensor, the stress tensor and the temperature, respectively. In the (experimental) literature the piezoelectric strain constant is usually denoted as d_{ijk}. These can be readily related to the e_{ijk} constants if the elastic compliances $s_{lmjk}^{E,T}$ of the material are known, then:

$$d_{ijk}^{T} = e_{ilm} s_{lmjk}^{E,T}$$

where summation over repeated indices is assumed [de Jong, M.; Chen, W.; Geerlings, H.; Asta, M.; Persson, K. A. A database to enable discovery and design of piezoelectric materials. *Sci Data* **2015**, *2*, 150053.]. For the trigonal system and point group 32 form of the piezoelectric tensor is following:

$$\begin{pmatrix} e_{11} & -e_{11} & 0 & e_{14} & 0 & 0 \\ 0 & 0 & 0 & 0 & -e_{14} & -e_{11} \\ 0 & 0 & 0 & 0 & 0 & 0 \end{pmatrix}$$

where piezoelectric e_{ijk}^{T} tensor has been written in shortened matrix Voigt notation. So, in the TMB crystal there are only two independent piezoelectric coefficient that have to be measured. From the work of de Jong, et al. and Fig. 6 therein it follows that in the chiral point group 32 the piezoelectric tensor $\|e_{ijk}^{T}\|$ largest values do not exceed 2.5 C/m^2 for any until now measured crystal. In our experiment we observed the reverse piezoelectric effect, the internal generation

of a mechanical strain was caused by externally applied electric field (E_3 || c-axis) used for measurement of dielectric response."

Considering the structure-property relationship it is reasonable to assume that the observed strongest piezoelectric resonance at 133 kHz for **TMB** single crystal is related to the lowest acoustic resonance with acoustic wave propagating within the direction perpendicular to the ***c***-axis. Taking into account that the resonance is broad one may suppose that it can be a mixture of several acoustic waves being in resonance with **TMB** crystal edges seen in Fig. 1 of the main manuscript. For the inducement of a piezoelectricity can be responsible the shearing strain that can deform the triangular base of bipyramids formed by Mn^{2+} and three Br1 atoms, however other charged units (organic parts) may be involved as well. It is well known that the procedure of measurements as well as calculations of piezoelectric crystal response is a difficult task and the results may differ due to material imperfections, defects, domains, etc.

In the main manuscript we add the following text and reference to ESI†:
"We simply observe that at certain crystal temperatures the used frequency of *AC* electric field matches perfectly with the mechanical crystal resonance (local $\varepsilon'(T)_{max}$) followed by the associated anti-resonance (local $\varepsilon'(T)_{min}$) as is clearly seen in Figure 5 (a). The additional information about the piezoelectric effect in this chiral space group *R*32 is described in ESI†."

3. Why the SHG intensity in Figure 9b is twice of that in Figure 9d? I assume the intensity should not change obviously by reversing the crystal to the light beam.

<u>Author note:</u>

This assumption is not correct in this case, reversing the crystal to the IR light beam causing SHG of light will have no effect for achiral crystal and chiral crystal illuminated only by linearly polarized light. Here we have illumination by LCP and RCP light that make a fundamental difference. This is a direct result which is expected when the same **TMB** purely chiral crystal is reversed by 180 degrees along the axis perpendicular to the c-axis and measured by SHG observation under constant rotation of a quarter wave retardation plate. This large difference is only observed for circularly polarized light and not for linearly polarized light. In the latter case the SHG response is the same irrespective of linear polarization azimuth (please see the Fig. 8 c and d). For the observed difference in SHG are responsible nonlinear optical processes due to coupling of electric and magnetic fields with a chiral structure, i.e. magneto-electric second order susceptibilities. The theory presented in ESI† and fitting of SHG-CD results is devoted entirely to explain these differences noticed by the Reviewer. Please also read the paper by Valev et al. Adv Mater 2013, 25, 2517–2534 where this problem is addressed.

4. "The measured photoluminescence quantum yield of TMB compound both in its powder and single crystal forms at room temperature is around 70%. Note that PLQY is significantly larger than that

reported previously.20" A PLQY of 70% is remarkably high for these hybrid perovskites, and the authors should explain why it is significantly improved compared with the previous report.

Author note:

In the case of PLQY results presented previously by Cai's group, the only information we can find in paper, that quantum yield was measured using the Edinburgh FLS920 fluorescence spectrometer. However, there is no information on how performance was measured - whether directly or indirectly. In the case of direct measurements, there are many factors that can affect the final result - the accuracy of absorption and emission measurements, as well as the selection of the standard. Our results were obtained using an integrating sphere. The results we obtain may therefore vary due to the measurement method we have chosen. Katoh R., et al. (DOI: 10.1021/jp807684m), pointed out that discrepancies in the quantum yield results for crystals may occur because the fluorescent properties of crystalline samples are sensitive to chemical impurities, structural defects and fluorescence reabsorption. Excitons migrating in the crystal are easily captured by impurities and structural defects, thereby causing fluorescence quenching of the host material. They also point out that many crystals can have either a quantum yield that is lower or higher than the powdered crystal. In our case, the quantum efficiency measurements for both the crystal and the powder are the same.

Additionally, the high PLQY 70 % for TMB crystal has been confirmed by a colleague from another laboratory on a calibrated apparatus. The high value of PLQY can be the result of lack of nonradiative deactivation channels around an excited Mn^{2+} ion. Such high values for Mn^{2+} ions have been already reported in literature: Zhang, J.; Sun, K-Q.; Zhang, Z-H.; Wang, R-C.; Lin, Z-H.; Lei, X-W.; Wang, Y-Y.; Ju, P.; and He, Y-C. Enhanced stability and tunable photoluminescence in Mn^{2+} -doped one-dimensional hybrid lead halide perovskites for high-performance white light emitting diodes. *RSC Adv*., **2023**, *13*, 19039.

With kindest regards,

Magdalena Rok with co-authors
Department of Ferroics and Semiconductors,
University of Wroclaw, Poland
